# MARCH2, a T cell specific factor that restricts HIV-1 infection

**DOI:** 10.1371/journal.ppat.1012330

**Published:** 2024-07-29

**Authors:** Supawadee Umthong, Uddhav Timilsina, Mary R. D’Angelo, Kyle Salka, Spyridon Stavrou

**Affiliations:** 1 Department of Microbiology and Immunology, Jacobs School of Medicine and Biomedical Sciences, University at Buffalo, Buffalo, New York, United States of America; 2 Department of Biochemistry and Microbiology, Faculty of Pharmaceutical Sciences, Chulalongkorn University, Bangkok, Thailand; Loyola University Chicago, UNITED STATES OF AMERICA

## Abstract

Membrane-associated RING-CH (MARCH) 2 is a member of the MARCH protein family of RING-CH finger E3 ubiquitin ligases that play important roles in regulating the levels of proteins found on the cell surface. MARCH1, 2 and 8 inhibit HIV-1 infection by preventing the incorporation of the envelope glycoproteins into nascent virions. However, a better understanding of the mechanism utilized by MARCH proteins to restrict HIV-1 infection is needed. In this report, we identify an amino acid in human MARCH2, absent in mouse MARCH2, critical for its antiretroviral function. Moreover, we map the domains of human MARCH2 critical for restricting as well as binding to the HIV-1 envelope glycoproteins. In addition, we demonstrate that MARCH2 is present inside nascent virions and reduces particle infectivity by blocking virus entry in a RING-CH-independent manner. Finally, we show that MARCH2 acts as an HIV-1 restriction factor only in primary CD4+ T cells and can prevent cell-to-cell transmission of HIV-1. Our findings reveal important new aspects of the antiviral mechanism utilized by human MARCH2 to restrict HIV-1 that have potential implications to all MARCH proteins with antiviral functions and their viral targets.

## Author summary

Recently, Membrane-associated RING-CH (MARCH) proteins have emerged as important antiviral factors that block virus infection by preventing viral envelope glycoprotein incorporation into newly formed virions in an ill-defined mechanism. Here, we examine in depth the role of MARCH2 during Human Immunodeficiency Virus type 1 (HIV-1) infection and show that it utilizes two mechanisms to inhibit HIV-1: 1) prevents the envelope glycoprotein incorporation into nascent virions in a RING-CH-dependent manner, 2) when incorporated in virions, it blocks virus-cell membrane fusion in a RING-CH independent manner. We also demonstrate that MARCH2 restricts HIV-1 only in CD4+ T cells and can prevent cell-to-cell transmission. In conclusion, our findings reveal new mechanistic aspects regarding the anti-HIV-1 function of MARCH2.

## Introduction

Retroviruses are a diverse family of viruses that infect numerous species and have the unique ability of integrating inside the host’s genome. Therefore, mammalian cells have developed defense mechanisms against them including proteins that can counteract every step of the retrovirus life cycle [1,2]. Among these host factors are the Membrane associated RING-CH (MARCH) family proteins, which are E3 ubiquitin ligases with important cellular functions [3,4]. MARCH proteins are highly conserved, found in all mammals, with mice and humans having 11 MARCH family members [5,6], and have critical immunomodulatory functions by regulating the levels of various immune receptors (e.g., CD86/B7.2, MHC-II etc.) found on the cell surface [3,4].

The functions of the MARCH protein family members extend beyond their role as immune receptor modulators. MARCH proteins have emerged as important antiviral factors that target a number of enveloped viruses [7]. Enveloped viruses contain a lipid bilayer on the surface of the viral particle, in which viral proteins critical for virus entry localize. In the case of Human Immunodeficiency Virus type 1 (HIV-1), the viral proteins found on the surface of the cell form a heterotrimer consisting of the surface (SU) glycoprotein gp120 and the transmembrane (TM) glycoprotein gp41 [8]. MARCH1, 2 and 8 exert their antiviral effect by preventing envelope glycoprotein incorporation into nascent virions for a number of viruses including HIV-1, Vesicular Stomatitis Virus (VSV), SARS-CoV-2, Influenza A virus and others [9–13]. Nevertheless, the mechanism of restriction has not been elucidated and seems not to be the same for all envelope glycoproteins [10,11,14,15]. Finally, MARCH8 is highly expressed in monocyte derived macrophages (MDMs), a cell type infected by HIV-1, and can inhibit HIV-1 infection at endogenous levels [9]. Thus, MARCH8 is an important factor in the cellular defense against HIV-1 infection in terminally differentiated myeloid cells.

While several studies have investigated the antiviral role of MARCH8, less is known about the role of the other MARCH proteins vis-à-vis retrovirus restriction. MARCH1 and 8 form a homologous pair, because of the high degree of sequence and structural homology, while MARCH2 is more similar to MARCH3, a MARCH protein with no known antiretroviral function [4,15,16]. We previously compared the human and murine orthologs of MARCH1, 2 and 8 and found that while mouse MARCH2 is about 95% homologous to human MARCH2, it has no antiviral function against retroviruses including HIV-1 and murine leukemia virus (MLV) [10], suggesting that MARCH2 acquired its antiviral function later on in the evolutionary scale.

In this report, we investigated the importance of MARCH2 during retrovirus infection. We demonstrate that Gly61, found in human MARCH2, but absent in mouse MARCH2, is critical for its anti-HIV-1 effect. Furthermore, we map the interaction between MARCH2 and HIV-1 envelope (Env). Moreover, we denote that only the long isoform of MARCH2 is antiretroviral, while the short isoform has no effect. We also show that MARCH2 incorporation in nascent virions is dependent on its interaction with the HIV-1 Env and inhibits virion intrinsic infectivity in a RING-CH independent manner by targeting cell-virus fusion. Finally, using primary cells and stable cell lines, we demonstrate that MARCH2 inhibits HIV-1 infection in a T cell-specific manner, reducing cell-to-cell transmission of HIV-1, while it has no antiviral effect in MDMs.

## Results

### Transcriptional regulation of *MARCH2*

**Fig 1:**
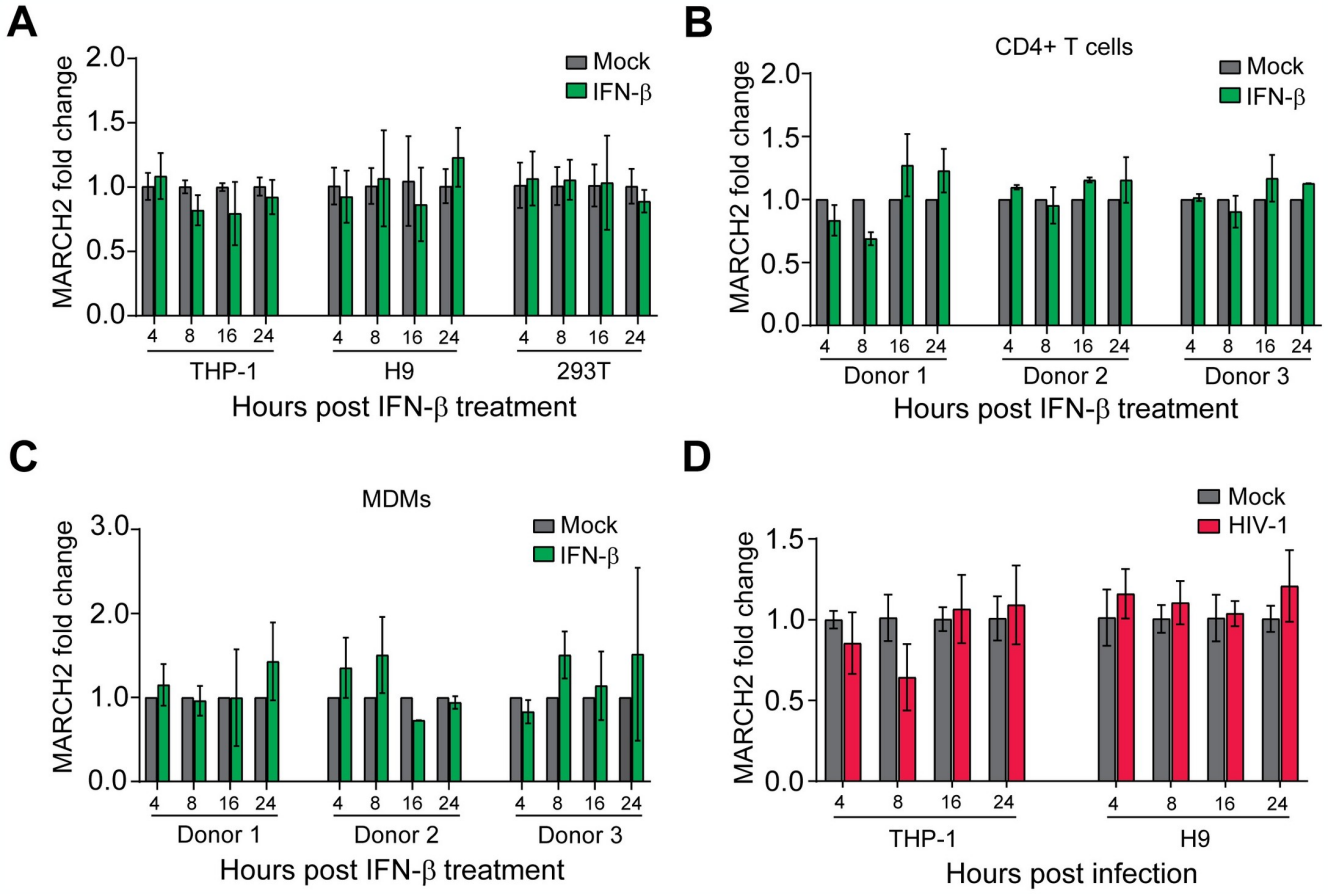
*MARCH2* gene expression is unaffected by type I IFN and HIV-1 infection. *MARCH2* fold expression change relative to mock normalized to *GAPDH* in **(A)** PMA-differentiated THP-1, H9, and 293T cells, **(B)** primary CD4+ T cells from three donors and **(C)** primary Monocyte Derived Macrophages (MDMs) from three donors treated with human IFN-β (500 units/ml) for 4 h, 8 h, 16 h, and 24 h. **(D)** *MARCH2* fold expression relative to mock normalized to GAPDH in PMA-differentiated THP-1 cells infected with HIV-1^JR-CSF^ and H9 cells infected with HIV-1^NL4-3^ at 4 h, 8 h, 16 h, and 24 h post infection (hpi). Mock indicates mock-treated (PBS). Graphs represent mean ± SD from n = 3 independent experiments.

Murine *March2* expression levels are not affected by type I interferon (IFN) stimulation or MLV infection [10]. In contrast, a previous report showed that human *MARCH2* is transcriptionally upregulated by IFNα, a type I IFN, in MDMs [15]. Thus, we examined the effect of type I IFN on *MARCH2* expression levels in H9 cells, a human T lymphocytic cell line, and phorbol 12-myristate 13-acetate (PMA)-differentiated THP-1 cells, a human monocytic cell line, both cell lines susceptible to HIV-1 infection, as well as 293T cells after treatment with human IFN-β (500 U/ml) (PBL Assay Science). We found that *MARCH2* RNA levels were unaffected upon IFN-β treatment in both cell lines tested (Fig 1A). As a positive control, we used *ISG15*, an IFN-stimulated gene [17] (S1A Fig). We repeated the aforementioned IFN-β treatment in primary CD4+ T cells and monocyte derived macrophages (MDMs) isolated from the buffy coats of three anonymous human donors (New York Blood Center) and found, similar to what we observed in H9 and THP-1 cells, *MARCH2* expression levels were unaffected by IFN-β treatment in primary CD4+ T cells and MDMs (Fig 1B and 1C). *ISG15*, an IFN-stimulated gene, served as our positive control (S1B and S1C Fig). A previous report found that HIV-1 induces the expression of *MARCH2* in 293T cells [18]. On the other hand, we have shown that MLV infection, did not affect mouse *March2* expression levels in either primary cells or in established cell lines [10]. Consequently, we examined the effect of HIV-1 infection on the RNA levels of *MARCH2* in H9 and THP-1 cells. We infected H9 cells and PMA-differentiated THP-1 cells with an X4-tropic (HIV-1^NL4-3^) and an R5-tropic (HIV-1^JR-CSF^) HIV-1 strain respectively and isolated RNA from the infected cells at various time points to measure *MARCH2* RNA levels. To ensure that our cells are infected, we initially measured HIV-1 *nef DNA* levels at different time points (S1D and S1E Fig). We found that HIV-1 infection had no effect on *MARCH2* expression in both cell lines tested (Fig 1D). Thus, we concluded that similar to murine *March2*, human *MARCH2* is not induced by IFN-β or HIV-1 infection.

### The N-terminal cytoplasmic tail of human MARCH2 is critical for its antiviral function

**Fig 2:**
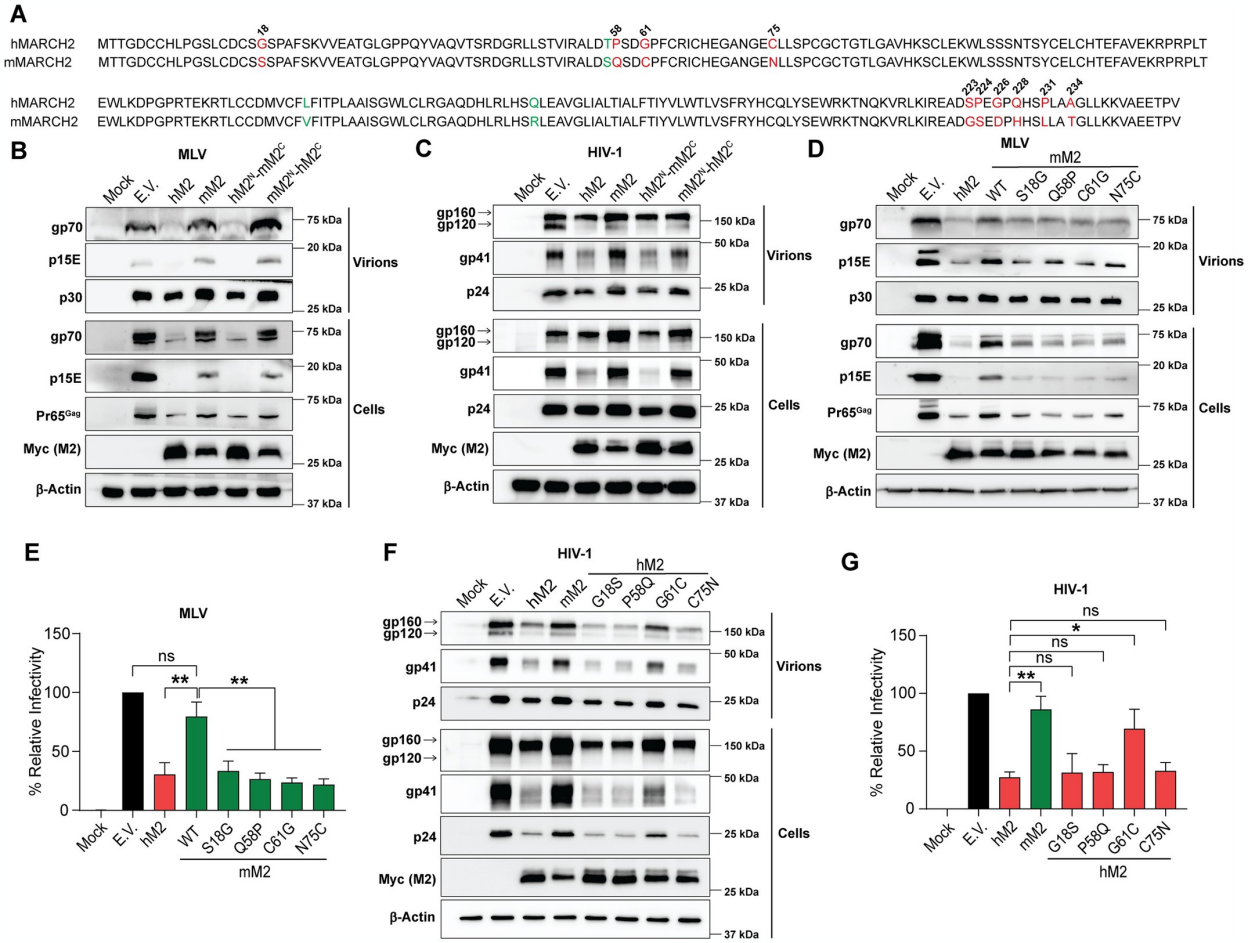
G61 residue on the N- terminal cytoplasmic tail of human MARCH2 is critical for its antiretroviral function. **(A)** Sequence alignment of human MARCH2 (hM2: NCBI Ref. NP_001356705.1) and mouse MARCH2 (mM2: NCBI Ref. NP_001239409.1). Differing amino acid residues are shown in red and their positions are indicated. In green are conservative changes in the amino acid sequence. **(B** and **C)** Western blots of cells and culture media from co-transfections of MLV (**B**) or HIV-1 (NL4-3) (**C**) in the presence of either wild type hM2 or mM2, cytoplasmic tails swapped M2 chimeras (hM2^N^-mM2^C^ or mM2^N^-hM2^C^) or empty vector (E.V.). **(D** and **E)** Substituting any 4 N-terminal cytoplasmic tail residues (S18, Q58, C61, and N75) of mM2 with those from hM2 renders mM2 antiviral. In **D**, western blots of cell and culture media of co-transfections of MLV in the presence of either wild type hM2 or mM2, mM2 mutants (S18G, Q58P, C61G, or N75C) or E.V. In **E**, infectivity assays of MLV containing a firefly luciferase reporter genome generated in the presence or absence of wild type hM2 or mM2 or the aforementioned mM2 mutants. Luciferase levels were measured 48 hpi and normalized to MLV p30 levels of the input virus. **(F** and **G)** G61 residue at the N-terminal cytoplasmic tail of hM2 is critical for its anti-HIV-1 function. In **F**, western blots of HIV-1 (NL4-3) co-transfections in the presence of either wild type hM2 or mM2, hM2 mutants (G18S, P58Q, G61C, or C75N) or E.V. In **G**, infectivity assays of NL4-3 Env pseudotyped luciferase reporter viruses generated in the presence of wild type hM2 or mM2, hM2 mutants (G18S, P58Q, G61C, or C75N) or E.V. Luciferase levels were measured 48 hpi and normalized to HIV-1 p24 levels of input virus. For **B**, **C**, **D** and **F**, representative immunoblotting results from n = 3 independent experiments are shown. Western blots were probed with anti-Myc, anti-gp70, anti-p15E, anti-Pr65^Gag^, anti-p24, anti-gp120, anti-gp41 and anti-β-Actin antibodies. For **E** and **G**, the percentage (%) of relative infectivity was determined with respect to virus produced in the presence of E.V. and all graphs represent mean ± SD from 3 independent experiments. Statistical significance was determined using one-sample t-test (two-tailed) when comparisons were performed with E.V. and unpaired t-test (two-tailed) was used for non-E.V. comparisons. ns, non-significant; *, *P* ≤ 0.05; **, *P* ≤ 0.01.

We previously showed that human MARCH2 inhibits HIV-1 infection, while mouse MARCH2 has no effect on retrovirus infection [10]. To investigate whether the difference in restriction phenotype could be clarified on the basis of genetic differences between the *MARCH2* genes from different species, we initially compared the amino acid sequences of the human and murine MARCH2. When we compared the human and mouse MARCH2 protein sequences, we observed that these orthologs shared 95% amino acid sequence identity with all sequence differences clustered within their N- and C- terminal cytoplasmic tails (Fig 2A). To determine which residues of human MARCH2 are important for its antiretroviral phenotype, we made a number of MARCH2 chimeras, in which we swapped the N-terminal half of human MARCH2 with that of mouse MARCH2 and vice versa (hMARCH2^N^–mMARCH2^C^ and mMARCH2^N^–hMARCH2^C^) (S2A Fig). Cells were co-transfected with either empty vector (E.V.), human MARCH2, mouse MARCH2 or the aforementioned MARCH2 chimeras along with either an MLV or HIV-1 molecular clone (NL4-3). Cells and media were harvested 48 hours post-transfection followed by western blots to determine the levels of the MLV (gp70/SU, p15E/p13E/TM) and HIV-1 (gp120/SU, gp41/TM) envelope glycoproteins. In the cell fractions, we found that the chimera containing the N-terminal end of human MARCH2 (hMARCH2^N^–mMARCH2^C^) resulted in the reduction of both MLV and HIV-1 envelope glycoprotein levels similar to that seen with human MARCH2. On the other hand, the chimera containing the N-terminal end of mouse MARCH2 (mMARCH2^N^–hMARCH2^C^) had no effect on the viral envelope glycoprotein levels similar to mouse MARCH2 (Fig 2B and 2C). Similarly, MLV and HIV-1 virions produced in the presence of either human MARCH2 or hMARCH2^N^–mMARCH2^C^ had significantly lower levels of gp70/p15E and gp120/gp41 respectively compared to virions produced in the presence of either E.V., mouse MARCH2 or mMARCH2^N^–hMARCH2^C^ (Fig 2B and 2C). Therefore, we concluded that the N-terminal end of human MARCH2 is critical for its antiviral effect.

There are four residues (18, 58, 61 and 75) in the N-terminal cytoplasmic tail of human and mouse MARCH2 that are radically different between the two orthologs and thus may cause significant changes in the MARCH2 protein structure and function (Fig 2A). While residue 18 is located far from the RING-CH domain, the other three (58, 61, and 75) are positioned proximal to it (S2A Fig). To determine which of these residues is/are important for the antiretroviral effect of human MARCH2, we made a number of human and mouse MARCH2 mutants, in which the amino acids found at the aforementioned sites in mouse MARCH2 were replaced by site directed mutagenesis to those found in human MARCH2 (S18G, Q58P, C61G, and N75C) and vice versa (G18S, P58Q, G61C, and C75N). Initially, we co-transfected 293T cells with an MLV infectious clone and either human MARCH2, mouse MARCH2, the mouse MARCH2 mutants (mMARCH2 S18G, mMARCH2 Q58P, mMARCH2 C61G, and mMARCH2 N75C) or E.V. We found that all mouse MARCH2 mutants tested, reduced gp70 and p15E levels in the cellular and viral fractions similar to the levels seen with human MARCH2 (Fig 2D), which was in agreement with our findings when measuring viral particle infectivity (Fig 2E). Therefore, we concluded that substituting any of these four sites in mouse MARCH2 with the corresponding amino acids found in human MARCH2 is sufficient to render mouse MARCH2 antiviral against MLV. To determine the residue(s) of human MARCH2 critical for its anti-HIV-1 function, we repeated the above experiment using an HIV-1 molecular clone (NL4-3) along with either wild type human or mouse MARCH2, the human MARCH2 mutants carrying the mouse MARCH2 amino acids at the same sites (hMARCH2 G18S, hMARCH2 P58Q, hMARCH2 G61C, and hMARCH2 C75N) or E.V. We found that only when human MARCH2 carried the amino acid of the mouse ortholog at position 61 (hMARCH2 G61C), did it lose its antiviral effect against HIV-1, as the gp120 and gp41 levels in the virus fraction were elevated (Fig 2F). In agreement with our western blot data, HIV-1 particle infectivity was significantly higher in virions produced in the presence of hMARCH2 G61C compared to those produced in the presence of wild type human MARCH2 (Fig 2G). Because of the importance of Gly61 of human MARCH2 on its anti-HIV-1 function, we used the mouse ortholog of MARCH2 carrying the reverse mutation (mMARCH2 C61G) to evaluate HIV-1 particle production. In contrast to what we observed with MLV, mMARCH2 C61G did not reduce HIV-1 infectivity, similar to what was observed with mouse MARCH2 (S2B Fig). Due to the proximity of residue 61 to the MARCH2 RING-CH domain, it is possible that hMARCH2 G61C has lost its antiviral activity, because Cys61 disrupts the RING-CH domain, thereby abrogating the E3 ubiquitin ligase activity of MARCH2. A previous report demonstrated that MARCH2 causes, via its E3 ubiquitin ligase activity, the ubiquitination and subsequent proteasomal degradation of the NF-κB essential modulator (NEMO) [19]. Therefore, to test if the MARCH2 E3 ubiquitin ligase activity is disrupted, we investigated the effect of hMARCH2 G61C on NEMO ubiquitination. As a negative control for our studies, we generated a MARCH2 RING-CH mutant by introducing a previously described mutation (W97A), which abrogates the E3 ubiquitin ligase activity of the RING-CH domain [15]. We co-transfected 293T cells with plasmids expressing NEMO, HA-tagged Ubiquitin along with either wild type hMARCH2, hMARCH2 W97A, hMARCH2 G61C or E.V. Cells were harvested, lysed and co-immunoprecipitations (coIPs) were performed pulling down for NEMO and probing for Ubiquitin. As expected, a smeared pattern associated with ubiquitination was observed for NEMO in the presence of either wild type hMARCH2 or hMARCH2 G61C, while in the presence of hMARCH2^W97A^ no ubiquitination of NEMO was observed (S2C Fig). Thus, we concluded that the G61C mutation does not affect the E3 ubiquitin ligase function of hMARCH2. In agreement with our findings, when we performed predictive molecular modeling using AlphaFold followed by visualization with iCn3D, we saw that substituting Gly61 with Cys did not affect the overall structure of hMARCH2 (S2D Fig).

In summary, our findings demonstrate that Gly61 at the N-terminal cytoplasmic tail of human MARCH2 is critical for its anti-HIV-1 function, yet it does not affect the MARCH2 E3 ubiquitin ligase activity. On the other hand, substituting any of the four residues in the mouse MARCH2 N-terminal cytoplasmic tail to those found in human MARCH2 rendered mouse MARCH2 antiviral against MLV.

### Naturally occurring MARCH2 polymorphisms restrict HIV-1 envelope

Single nucleotide variants (SNVs) in cellular restriction factors can dramatically influence their ability to counteract HIV-1 infection. Previous reports have identified SNVs in TRIM5α and APOBEC3D that affect their anti-HIV-1 function [20–22]. To examine for SNVs in the *MARCH2* locus, we accessed the 1000 Genome Project Phase III genotypes for the coding region of the *MARCH2* gene (n = 5008). We investigated for missense SNVs that occur at high frequency and would lead to radical changes in the amino acid sequence of MARCH2 protein. Two SNVs that fit our search criteria were an alanine changed to a threonine at position 54 (A54T/rs1133893) and an arginine changed to a proline at position 219 (R219P/rs34099346). The minor allele frequency of A54T was the highest in European and American populations (0.2922 and 0.272 respectively) and that of R219P was the highest in South Asian (0.174), American (0.167) and European (0.167) populations (S1 Table). To determine the effect of these SNVs on the antiviral function of MARCH2, we generated NL4-3 Env pseudotyped luciferase reporter HIV-1 viruses in the presence of varying amounts of wild type MARCH2 or MARCH2 carrying either of the two SNVs (MARCH2 A54T and MARCH2 R219P). We found that virions generated with MARCH2 carrying the R219P SNV, in all concentrations tested, led to lower gp120 and gp41 levels compared to those produced in the presence of wild type MARCH2 (S3A Fig). To determine if the decreased incorporation of gp120 and gp41 in the nascent virions affected virus infectivity, we infected U373-MAGI-CXCR4 cells and measured luminescence levels 48 hours later. We found that compared to wild type MARCH2, MARCH2 carrying the R219P SNV (and the A54T SNV at high amounts) was more potent in inhibiting HIV-1 particle infectivity in agreement with our western blot data (S3B Fig). Thus, the R219P/rs34099346 MARCH2 polymorphisms imparts a gain-of-function effect on the antiviral function of MARCH2.

### Mapping the domains of MARCH2 responsible for its anti-HIV-1 function

**Fig 3:**
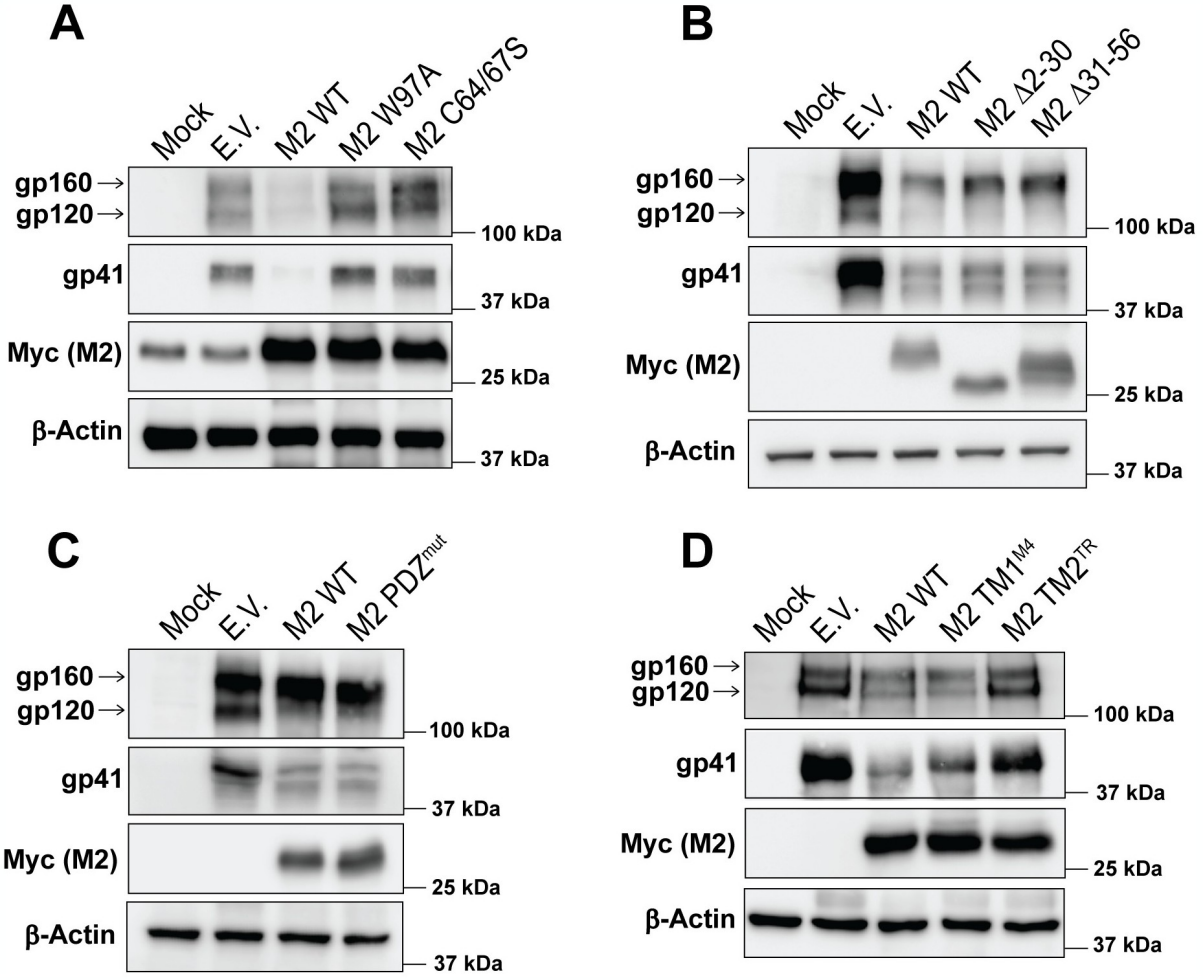
The RING-CH domain and the TM2 domain of human MARCH2 are important for the degradation of HIV-1 envelope glycoprotein. 293T cells were co-transfected with an HIV-1 infectious clone (NL4-3) along with either empty vector (E.V.), wild type (WT) MARCH2 (M2) or the following M2 mutants: **(A)** M2 RING-CH domain mutants (M2 W97A and M2 C64/67S), **(B)** M2 with deletions of N-terminal amino acid residues 2–30 (M2 Δ2–30) or 31–56 (M2 Δ31–56), **(C)** M2 with mutations in the PDZ binding motif (M2 PDZ^mut^) and **(D)** M2 with either N-terminal (TM1) or C-terminal (TM2) swapped with those of human MARCH4 (M2 TM1^M4^) and human transferrin receptor (M2 TM2^TR^) respectively. Cells were harvested 48 h post transfection, and proteins were analyzed by immunoblotting using anti-Myc, anti-p24, anti-gp120, anti-gp41 and anti-β-Actin antibodies. For **A**- **D**, representative immunoblotting results from n = 3 independent experiments are shown.

**Fig 4:**
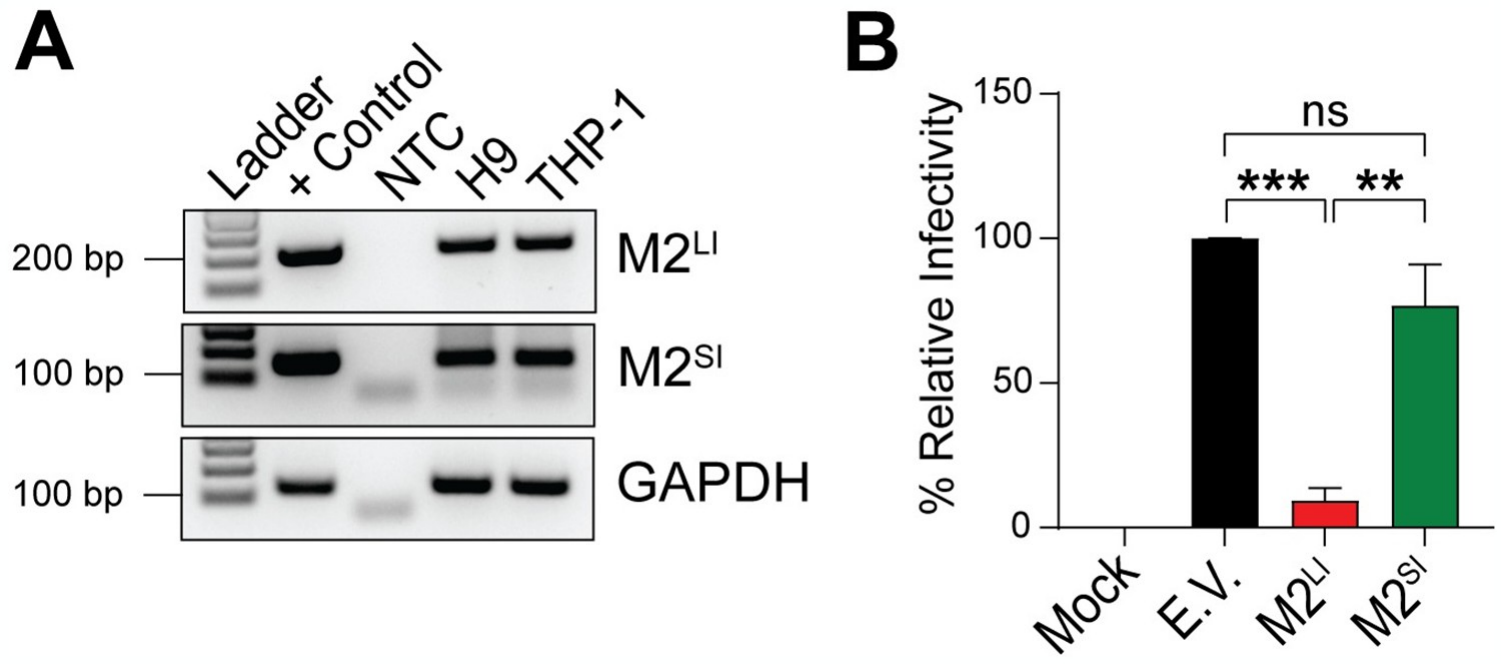
Only the canonical long isoform of MARCH2 has anti-HIV-1 function. **(A)** Both isoforms of human MARCH2 (M2), M2 long isoform (M2^LI^) and M2 short isoform (M2^SI^) are expressed in H9 and THP-1 cells. M2 isoform specific products were amplified by PCR from H9 and THP-1 cDNAs followed by agarose gel analysis. GAPDH was used as a loading control and M2 plasmid DNA was used as positive control. **(B)** M2^LI^ reduces HIV-1 infectivity. NL4-3 Env pseudotyped luciferase reporter viruses were generated in the presence of either M2^LI^, M2^SI^ or empty vector (E.V.). Pseudoviruses were used to infect U373-MAGI-CXCR4 cells, luciferase levels were measured 48 hpi and normalized to HIV-1 p24 levels of the input virus. The percentage (%) of relative infectivity was determined with respect to virus produced in the presence of E.V. In **A**, representative gel images from n = 2 independent experiments are shown. In **B**, graphs represent mean ± SD from 3 independent experiments. Statistical significance was determined using one-sample t-test (two-tailed) when comparisons were performed with E.V. and unpaired t-test (two-tailed) was used for the comparison between M2^LI^ and M2^SI^. ns, non-significant; **, *P* ≤ 0.01; ***, *P* ≤ 0.001. (no template control, NTC; + Control, positive control).

MARCH2 has two transmembrane (TM) domains and two cytoplasmic tails, an N-terminal tail, in which the RING-CH domain is located, and a C-terminal tail, which contains the PDZ binding motif (S2A Fig) [5,23]. To investigate the role of these domains on the MARCH2-mediated anti-HIV-1 activity, we generated a series of constructs with mutations in the aforementioned domains. Initially, we confirmed that the mutations we introduced in MARCH2 did not affect its localization to cellular membranes. As the MARCH2 available antibodies are not suitable for flow cytometry, we performed western blots on lysates of membrane bound proteins isolated from 293T cells transfected with either wild type or mutant MARCH2 expression plasmids. We found that all MARCH2 mutants tested, localized to cellular membranes resembling wild type MARCH2 (S4A Fig). To examine the role of the RING-CH domain on the anti-HIV-1 activity of MARCH2, we used the previously described MARCH2 RING-CH domain mutant (MARCH2^W97A^) and a second MARCH2 RING-CH domain mutant, in which Cys64 and Cys67 have been mutated to Ser (MARCH2^C64/67S^) and has been shown to have no E3 Ubiquitin ligase activity [24]. We observed that in the presence of both MARCH2^W97A^ and MARCH2^C64/67S^, the cellular levels of gp120 and gp41 were similar to those seen in the presence of E.V. (Fig 3A). When using MARCH2 constructs with deletions at the distal end of the N-terminal cytoplasmic tail upstream of the RING-CH domain (Δ2–30 and Δ31–56 amino acids), we noticed that MARCH2Δ2–30 and MARCH2Δ31–56 reduced gp120 and gp41 levels similar to those seen with wild type MARCH2 (Fig 3B). Likewise, mutations in the PDZ binding motif (243-ETVA-246 to 243-AAAA-246) did not affect the ability of MARCH2 to reduce gp120 and gp41 levels (Fig 3C). To investigate the role of the MARCH2 TM domains on retrovirus restriction, we generated a series of mutants, in which we substituted the TM domains of MARCH2 with those of MARCH4 that has no anti-HIV-1 activity [15]. Interestingly, we found that the exchange of the second TM domain of MARCH2 with that from MARCH4 affected protein expression (S4B Fig). Therefore, we replaced the second TM domain of MARCH2 with that of the human transferrin receptor (TR). We initially verified that swapping the TM domains of MARCH2 did not affect MARCH2 presence in cellular membranes (S4C Fig). In the case of the second TM domain MARCH2 mutant, we also performed immunofluorescence (IF) and found that its subcellular localization was similar to that of wild type MARCH2 (S4D Fig). Next, we found that mutating the second TM domain of MARCH2 rendered it unable to reduce the gp120 and gp41 levels (Fig 3D). The importance of the TM domains on the antiviral effect of MARCH2 is further supported by our studies on the two isoforms of MARCH2, which are products of alternative splicing. The canonical long isoform of MARCH2 (*March2-001*) has two TM domains, while the non-canonical shorter isoform (*March2-002*) lacks both TM domains (Δ125–194) (S5 Fig). We initially verified the expression of both isoforms in H9 and THP-1 cells by PCR (Fig 4A). Subsequently, we generated NL4-3 Env pseudotyped luciferase reporter viruses in the presence of either MARCH2 isoform and measured particle infectivity of the generated viruses. We found that viral particle infectivity was only reduced in the presence of the long MARCH2 isoform, while the short MARCH2 isoform had no effect (Fig 4B). In conclusion, our findings show that the RING-CH domain and the TM domains of MARCH2 are critical for its antiviral function.

### MARCH2 interacts with HIV-1 gp41

**Fig 5:**
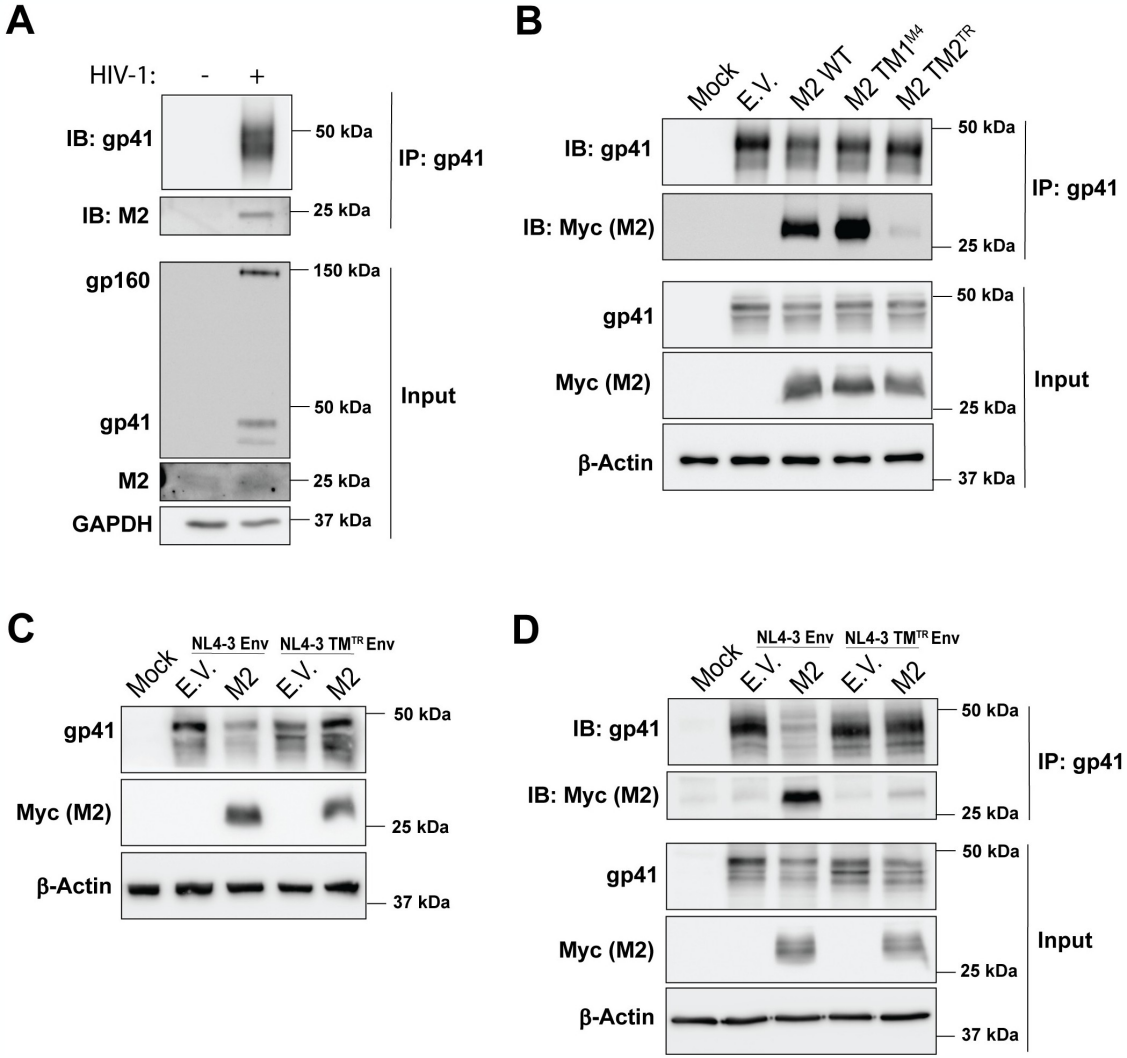
MARCH2 and HIV-1 gp41 interaction is mediated by their TM domains. **(A)** Endogenous MARCH2 (M2) interacts with HIV-1 gp41. Cell lysates from HIV-1^NL4-3^ infected H9 cells were immunoprecipitated (IPed) with an anti-HIV-1 gp41 antibody followed by western blots probing with anti-M2, anti-HIV-1 gp41 and anti-GAPDH antibodies. **(B)** The C- terminal TM domain (TM2) of M2 is critical for its interaction with HIV-1 gp41. CoIPs of cell lysates of 293T cells co-transfected with an HIV-1 infectious clone along with either empty vector (E.V.), M2 WT or M2 TM domain mutants (M2 TM1^M4^, M2 TM2^TR^). Cells were harvested, lysed and immunoprecipitated with an anti-HIV-1 gp41 antibody followed by western blots probing with anti-Myc (M2), anti-HIV-1 gp41 and anti-β-Actin antibodies. **(C)** HIV-1 gp41 TM domain is required for M2-mediated restriction. 293T cells were co-transfected with plasmids encoding either NL4-3 Env or mutant NL4-3 Env, in which the TM domain has been swapped with that of transferrin receptor, (NL4-3 TM^TR^ Env) along with either E.V. or M2. Cells were lysed followed by western blot analysis with anti-HIV-1 gp41, anti-Myc (M2) and anti-β-Actin antibodies. **(D)** The TM domain of HIV-1 gp41 is required for its interaction with M2. Cells were transfected as in **C**, lysed and immunoprecipitations were performed with an anti-HIV-1 gp41 antibody followed by western blots probing with anti-Myc (M2), anti-HIV-1 gp41 and anti-β-Actin antibodies. For **A- D**, representative immunoblotting results from n = 3 independent experiments are shown.

MARCH proteins form complexes with their cellular targets [10,12,25,26]. However, all previous studies have been performed in the context of overexpression [9,10,12]. Therefore, we set out to determine if HIV-1 Env interacts with endogenously expressed MARCH2 in immune cells. Consequently, we performed coIPs using cell lysates from HIV-1 infected H9 cells, which express endogenous levels of MARCH2 (Fig 4A). We found that endogenous MARCH2 coIPed with HIV-1 gp41 during infection (Fig 5A). To better understand how MARCH2 and HIV-1 gp41 interact, we co-transfected 293T cells with NL4-3 and either E.V., wild type MARCH2 or the TM mutant MARCH2 constructs mentioned above (Fig 3D). To ensure that MARCH2 did not reduce the HIV-1 envelope glycoprotein levels impacting our coIP studies, we used low concentrations of the MARCH2 expression plasmids. We then performed coIPs pulling down for HIV-1 gp41 and probing for MARCH2. We found that only wild type MARCH2 and MARCH2, in which the first TM domain is mutated, interacted with HIV-1 gp41 (Fig 5B). Swapping the second TM domain of MARCH2 to that of human TR abolished that interaction (Fig 5B). Thus, our findings show that MARCH2 interacts with the HIV-1 envelope glycoproteins via the second TM domain. To better understand the interaction between HIV-1 gp41 and MARCH2, we investigated the role of the TM domain of gp41 by generating a mutant HIV-1 envelope expressing plasmid, in which the gp41 TM domain is replaced with that of human TR (NL4-3 TM^TR^ Env). To ensure that mutating the TM domain of HIV-1 gp41 did not affect gp41 localization at the plasma membrane, we transfected 293T cells with either the wild type NL4-3 Env or the NL4-3 TM^TR^ Env followed by flow cytometry using an anti-HIV-1 antibody (VRCO1) [27]. We found similar cell surface expression levels for both Env constructs (S6 Fig) indicating that mutating the TM domain of HIV-1 gp41 did not alter the gp41 plasma membrane localization. We then examined whether NL4-3 TM^TR^ Env is resistant to MARCH2 restriction by co-transfecting 293T cells with either NL4-3 Env or NL4-3 TM^TR^ Env along with MARCH2 followed by western blots. We found that NL4-3 TM^TR^ Env was resistant to MARCH2-mediated inhibition (Fig 5C). We then performed coIPs using cell lysates from 293T cells co-transfected with either NL4-3 Env or NL4-3 TM^TR^ Env expression plasmids along with MARCH2. We noticed that mutating the TM domain of HIV-1 gp41 potently inhibited the interaction between MARCH2 and gp41 (Fig 5D). Taken together, our findings show that endogenous MARCH2 and HIV-1 gp41 interact and their association is mediated by their TM domains.

### MARCH2 is incorporated in nascent viral particles in a TM-dependent manner

**Fig 6:**
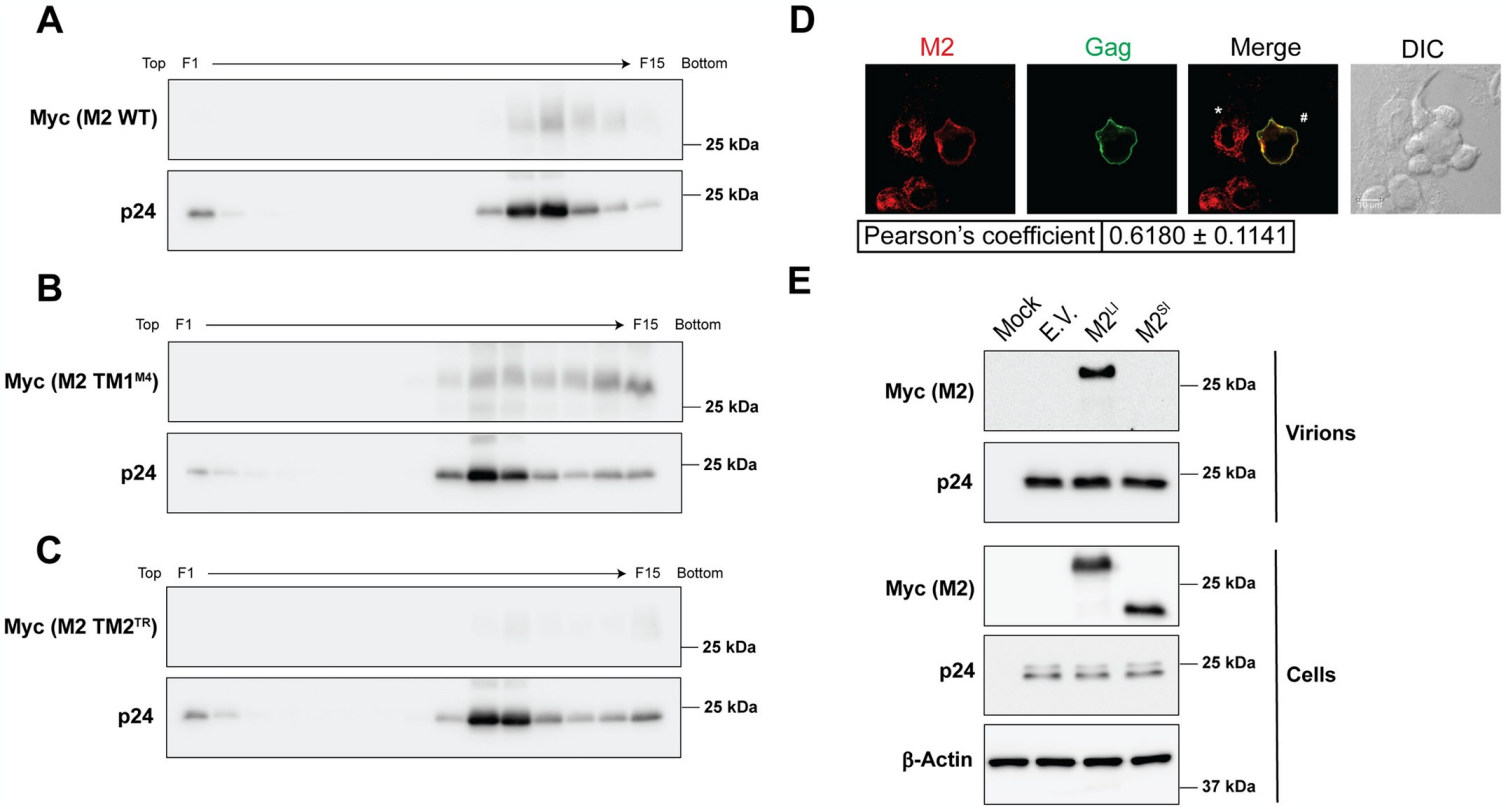
MARCH2 is incorporated in HIV-1 virions in a transmembrane (TM)-dependent manner. **(A**-**C)** C-terminal TM domain (TM2) of MARCH2 (M2) is critical for M2 incorporation in nascent HIV-1 particles. HIV-1 particles produced in the presence of either wild type (WT) M2 or MARCH2 TM domain mutants (M2 TM1^M4^, M2 TM2^TR^). Culture supernatants were harvested 48 h post transfection, concentrated through 30% sucrose cushion, and subjected to OptiPrep (5 to 20%) density gradient fractionation. Proteins were recovered from the fractions by TCA precipitation and analyzed by western blots probing with anti-Myc (M2) and anti-p24 antibodies. **(D)** M2 co-localizes with HIV-1 Gag at the cell surface. AD293 cells co-transfected with plasmids expressing HIV Gag-iGFP and M2 WT were subjected to immunostaining. The table at the bottom shows Pearson’s correlation coefficient (mean ± SD) for co-localization between M2 and HIV-1 Gag (Gag) calculated from 5 independent experiments. **(E)** The M2 short isoform (M2^SI^) that lacks the TM domain is not incorporated in nascent HIV-1 virions. Virus from the culture media and cell lysates from 293T cells transfected with an HIV-1 infectious clone and either empty vector (E.V.), M2^SI^ or the M2 long isoform (M2^LI^) were analyzed by western blots probing with anti-Myc (M2), anti-p24 and anti-β-Actin. For **A-C** and **E** representative of n = 3 independent experiments are shown. In **D**, representative deconvolved single Z-section images are shown. (Fraction 1, F1; Fraction 15, F15).

We established above that MARCH2 and the HIV-1 envelope glycoproteins interact via their TM domains. Thus, we speculated that the TM-mediated interaction between MARCH2 and the HIV-1 envelope glycoproteins may facilitate the incorporation of MARCH2 into nascent virions. Therefore, we co-transfected 293T cells with NL4-3 along with either wild type MARCH2 or the TM mutant MARCH2 constructs mentioned above (Fig 3D). Culture supernatants were harvested 48 hpi and pelleted through 30% sucrose cushion. In order to distinguish HIV-1 particles from exosomes/macrovesicles, the pelleted material was then subjected to an OptiPrep (5 to 20% iodixanol gradients) (Stemcell Technologies) velocity gradient [28–30] and the subsequent fractions were subjected to immunoblotting. We clearly detected wild type and TM1 mutant MARCH2 [which interact with HIV-1 envelope (Fig 5B)] in the HIV-1 p24 enriched fractions (Fig 6A and 6B), while TM2 mutant MARCH2 [which failed to interact with HIV-1 gp41 (Fig 5B)] was not detected in the HIV-1 p24 enriched fractions (Fig 6C). Interestingly, in the case of wild type MARCH2, HIV-1 p24 was present in 6 fractions, while 7 fractions were positive for HIV-1 p24 in the mutant MARCH2 conditions (Fig 6A–6C). We speculate the reason for this discrepancy is likely technical during the collection of fractions.

We showed above that MARCH2 is found in membranous intracellular structures (S4D Fig), yet our findings clearly demonstrate that it is incorporated in budding viral particles. Thus, it is not clear how MARCH2 is incorporated in nascent virions as it does not localize at the sites of HIV-1 virion assembly, the plasma membrane. Consequently, we hypothesized that HIV-1 infection alters MARCH2 subcellular localization resulting in its presence at the plasma membrane. To examine this, we co-transfected AD293 cells with the HIV Gag-iGFP molecular clone along with MARCH2 followed by IF. Similar to our findings shown above (S4D Fig), in cells expressing only MARCH2 (indicated by *), MARCH2 was detected in intracellular punctuate structures (Fig 6D). Interestingly, cells expressing HIV-1 Gag and MARCH2 (indicated by #), MARCH2 co-localized with HIV-1 Gag on the cell surface, presumably at the sites of virion assembly. Thus, our data suggest that during infection MARCH2 translocates to the plasma membrane, where HIV-1 virions assemble, thereby becoming incorporated in nascent virions.

Knowing that the MARCH2 interaction with the HIV-1 gp41 and virion incorporation is dependent on its second TM domain, we hypothesized that the shorter non-canonical MARCH2 isoform that lacks the TM domains (S5 Fig) would not be incorporated into nascent virions. By performing western blots on HIV-1 virions produced in the presence of the two MARCH2 isoforms, we found that only the TM-containing longer isoform of MARCH2 is packaged into HIV-1 virions (Fig 6E). Therefore, we conclude that MARCH2 is present in viral particles and its presence in HIV-1 virions is dependent on its interaction with the HIV-1 envelope glycoproteins via its second TM domain.

### MARCH2 present in HIV-1 virions blocks particle infectivity in a RING-CH-independent manner

**Fig 7:**
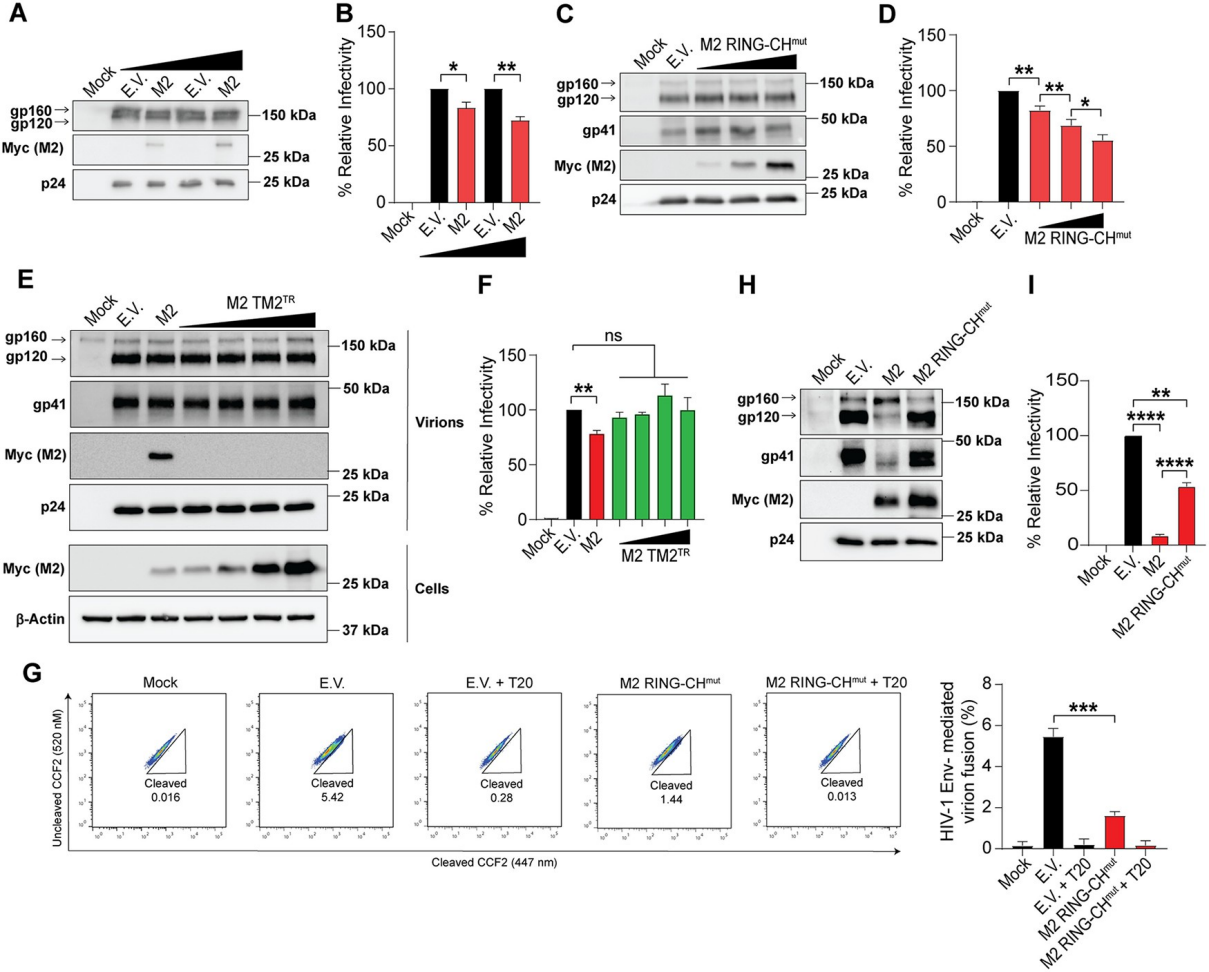
Virion-incorporated MARCH2 blocks HIV-1 particle entry by targeting virus-cell fusion in a RING-CH-independent manner. **(A** and **B)** Low levels of MARCH2 (M2) have no effect on envelope (Env) glycoprotein levels yet still reduce HIV-1 particle infectivity. HIV-1 particles produced in the presence of either low amounts of M2 or empty vector (E.V.) were analyzed by western blots. In **B**, HIV-1 from **A** was normalized for gp120 and used to infect TZM-bl cells; luciferase levels were measured 48 hpi and normalized to HIV-1 p24 levels of the input virus. **(C** and **D)** M2 RING-CH mutant reduces HIV-1 particle infectivity. NL4-3 Env pseudotyped luciferase reporter viruses were produced in either increasing concentrations of RING-CH domain mutant of MARCH2 (M2 RING-CH^mut^) or eGFP-N1 (serving as E.V.). Culture media were harvested and either analyzed by (**C**) western blots or (**D**) used to infect U373-MAGI-CXCR4 cells, luciferase levels were measured 48 hpi and normalized to HIV-1 p24 levels of the input virus. **(E** and **F)** M2 TM2^TR^ has no effect on gp120 and gp41 levels and is not incorporated in nascent virions. NL4-3 Env pseudotyped luciferase reporter viruses were produced in either increasing concentrations of M2 TM2^TR^, low levels of wild type M2 or E.V. (**E**) western blots and (**F**) infectivity assays as described in **C** and **D**. **(G)** MARCH2 inhibits HIV-1 Env-mediated fusion. H9 cells were infected with p24- normalized HIV-1 produced in the presence of either M2 RING-CH^mut^ or E.V. Fusion inhibitor enfuvirtide (T20) served as a negative control. Cells were stained with CCF2-AM and then subjected to FACS. Fusion efficiency was quantified as the percentage of cleaved CCF2. Representative FACS plots and graphs representing mean ± SD of % cleaved CCF2 positive cells from n = 3 independent experiments are shown. (**H** and **I**) M2-mediated inhibition of envelope incorporation in nascent HIV-1 virions and inhibition of virus entry by M2 inhibit HIV-1 particle infectivity in an additive manner. NL4-3 Env pseudotyped luciferase reporter viruses were generated in the presence of E.V., wild type M2 or M2 RING-CH^mut^. In **H**, western blots and in **I**, infectivity assays as described in (**C**) and (**D**). Luciferase levels were measured 48 hpi and normalized to HIV-1 p24 levels of the input virus. In **B**, **D**, **F** and **I**, the percentage (%) of relative infectivity was determined with respect to virus produced in the presence of E.V. For **A**, **C, E** and **H**, representative western blots from n = 3 independent experiments are shown probing with anti-gp120, anti-gp41, anti-p24, anti-Myc (M2) and anti-β-Actin antibodies. Graphs in **B**, **D**, **F** and **I** represent mean ± SD from 3 independent experiments. Statistical significance was determined using one-sample t-test (two-tailed) when comparisons were performed with E.V. in **B**, **D**, **F** and **I** and unpaired t-test (two-tailed) between non E.V. conditions in **D** and **I** and for any comparisons in **G**. ns, non-significant; *, *P* ≤ 0.05; **, *P* ≤ 0.01; ***, *P* ≤ 0.001; ****, *P* ≤ 0.0001.

We demonstrate above that MARCH2 is incorporated in nascent HIV-1 virions. This observation led us to ask if the virion-incorporated MARCH2 would affect viral particle infectivity. To test this, we co-transfected 293T cells with NL4-3 in the presence of low levels of MARCH2 so that MARCH2 has little to no effect on viral envelope levels but is still incorporated into HIV-1 virions. After transfection, we collected culture supernatants and confirmed by western blot and densitometry that MARCH2 was incorporated in virions and viral gp120 levels were unaffected by MARCH2 (Figs 7A and S7A). Subsequently, we infected TZM-bl cells with equal amount of gp120 and luciferase levels normalized to p24 to determine particle infectivity. We found that MARCH2 incorporated in nascent virions reduced HIV-1 particle infectivity in a dose dependent manner (Fig 7B). Nevertheless, the decrease observed was modest, which we account to the use of very low levels of MARCH2 to avoid confounding effects on envelope incorporation. Yet, we were still concerned that MARCH2 may affect the envelope levels to a degree that cannot be detected by western blot or ELISA. Thus, we resorted to an alternative approach; we showed above that RING-CH mutant MARCH2 did not affect the cellular levels of the HIV-1 envelope glycoproteins (Fig 3A). Therefore, RING-CH mutant MARCH2: (1) does not affect the incorporation of HIV-1 envelope glycoproteins in nascent virions, and (2) has intact TM domains and thus should be able to become incorporated into newly generated HIV-1 virions. Consequently, we generated NL4-3 Env pseudotyped luciferase reporter viruses with increasing amounts of the RING-CH mutant MARCH2. First, we confirmed by western blot and densitometry that RING-CH mutant MARCH2 was incorporated in newly produced HIV-1 virions in a dose dependent manner and did not affect the envelope glycoprotein levels in our viral particles (Figs 7C and S7B). Next, we performed infectivity assays using U373-MAGI-CXCR4 cells and observed that HIV-1 virions produced in the presence of the RING-CH mutant MARCH2 displayed a dose-dependent decrease in particle infectivity, when compared to those produced in the presence of E.V. (Fig 7D). Subsequently, we examined the effect of MARCH2, in which the second TM domain has been replaced with that of the transferrin receptor, MARCH2 TM2^TR^, and no longer interacts with the HIV-1 Env or is incorporated in nascent virions (Figs 5B and 6C). We generated HIV-1 particles in the presence of increasing amounts of MARCH2 TM2^TR^ followed by western blots and densitometry. As shown above, we found that unlike wild type MARCH2 or RING-CH mutant MARCH2, MARCH2 TM2^TR^ did not reduce gp120 and gp41 levels and was not incorporated in nascent virions (Figs 7E and S7C). As expected, when performing infectivity assays in U373-MAGI-CXCR4 cells, we observed no effect in particle infectivity (Fig 7F). Finally, as the transmembrane domains of mouse MARCH2 are identical to those of human MARCH2, we examined whether mouse MARCH2 can also be incorporated in nascent virions in a RING-CH independent manner. As expected, we found, similar to human MARCH2, mouse MARCH2 was incorporated in nascent virions in a RING-CH independent manner (S7D Fig). The above findings show that MARCH2 incorporated in nascent virions affects HIV-1 particle infectivity independent of its RING-CH domain.

As MARCH2 interacts with the HIV-1 envelope glycoproteins via TM-TM interactions, we hypothesized that MARCH2 may interfere with virus-cell fusion during entry. To address the effect of MARCH2 on virus-cell fusion, we utilized the beta-lactamase-Vpr (BlaM-Vpr) fusion assay. In this assay, BlaM-Vpr chimeric protein is incorporated in virions and is subsequently transported into the cytoplasm of target cells as a result of virus-cell fusion, where it enzymatically cleaves CCF2, a fluorescent dye that has been preloaded into cells, and its cleavage can be detected by flow cytometry [31]. HIV-1 virions containing BlaM-Vpr were produced in the presence of either E.V. or the RING-CH mutant MARCH2. We measured p24 levels by ELISA and infected H9 cells pre-loaded with CCF2 with equal amounts of p24 followed by flow cytometry to measure cleaved CCF2 levels. As a negative control we used T20, an HIV-1 fusion inhibitor [32]. We observed lower levels of fusion when infecting with viral particles produced in the presence of the RING-CH mutant MARCH2 compared to the levels seen with virions produced in the presence of E.V. (Fig 7G). The addition of T20, as expected, abrogated virus-cell fusion (Fig 7G). Thus, MARCH2 blocks HIV-1 infectivity utilizing two mechanisms, inhibition of envelope incorporation in nascent virions and disruption of virus entry by hindering virus-cell fusion. To determine the contribution of these two antiretroviral functions on the MARCH2 anti-HIV-1 effect, we generated NL4-3 Env pseudotyped luciferase reporter viruses in the presence of either E.V., wild type MARCH2 or RING-CH mutant MARCH2. We first verified by western blot and densitometry that gp120 and gp41 levels were unaffected by the MARCH2 RING-CH mutant when compared to E.V. control (Figs 7H and S7E) and that both wild type and RING-CH mutant MARCH2 were similarly incorporated in purified HIV-1 virions (Fig 7H). When measuring virus infectivity, we observed that virus produced in the presence of wild type MARCH2 was about 5-fold less infectious than virus produced in the presence of the RING-CH mutant MARCH2 (Fig 7I). Yet, infectivity of virions produced in the presence of RING-CH mutant MARCH2 was lower than that of virions produced in the presence of E.V. (Fig 7I). In summary, our findings show that MARCH2 uses two mechanisms to block HIV-1 infectivity. MARCH2 present in nascent virions can reduce particle infectivity by interfering with virus-cell fusion in a RING-CH independent manner and in the producer cells, MARCH2 blocks envelope incorporation in nascent virions.

### Endogenous MARCH2 restricts HIV-1 replication in a T cell specific manner

**Fig 8:**
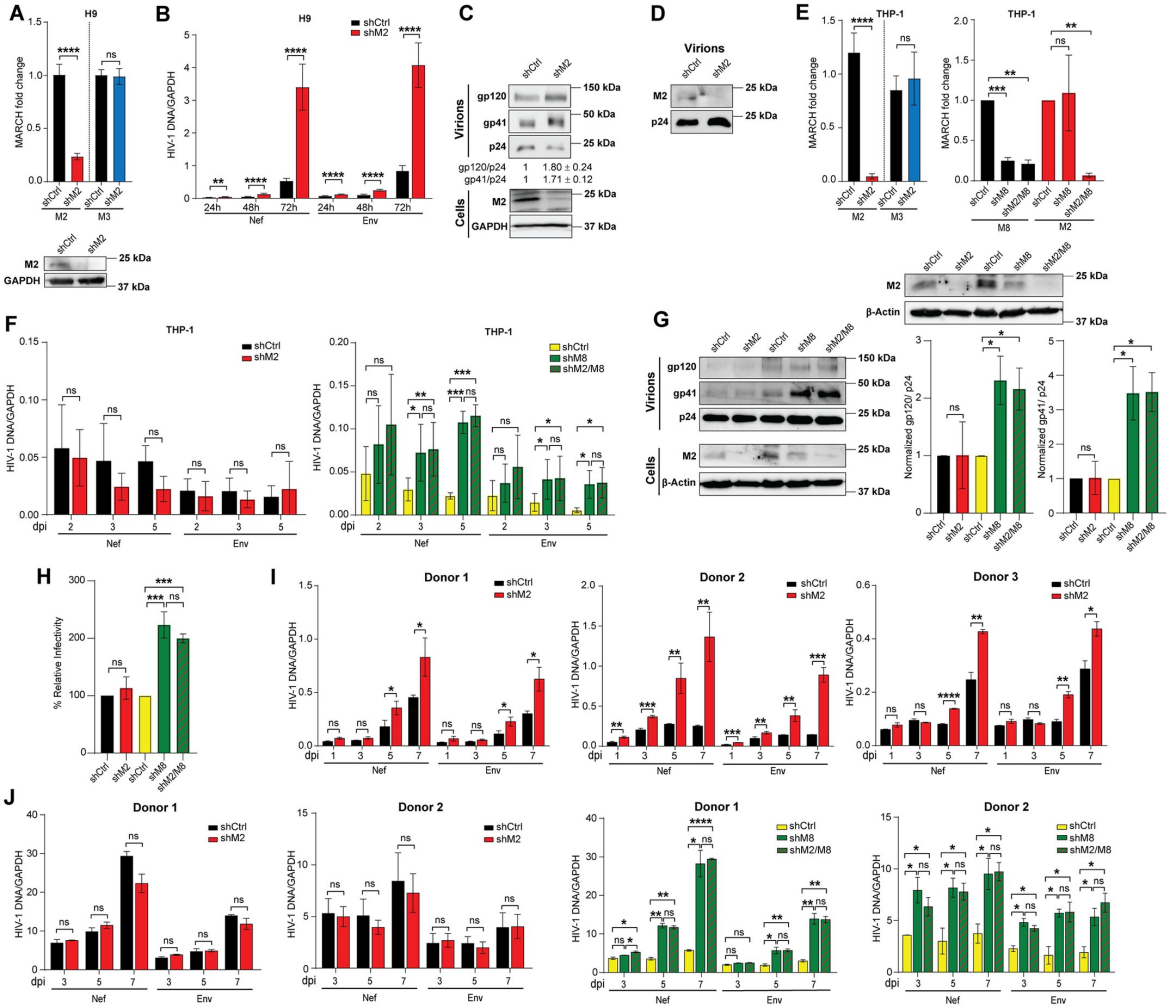
MARCH2 restricts HIV-1 replication in a T cell specific manner. **(A**) MARCH2 (M2) knockdown verification in H9 cells knocked down with a *M2* specific (shM2) or a scramble control (shCtrl) shRNA. Fold expression change of human *M2* and *MARCH3 (M3)* transcripts in M2 depleted H9 cells relative to shCtrl treated cells and normalized to GAPDH is shown. Bottom panels show western blots of endogenous M2 proteins in shCtrl- or shM2- treated H9 cells. GAPDH serves as loading control. **(B)** Endogenous M2 reduces HIV-1 replication in H9 cells. Wild type (shCtrl) or M2 depleted (shM2) H9 cells were infected with HIV-1^NL4-3^ and harvested at the indicated time points. HIV-1 *nef* and *env* DNA levels were determined by RT-PCR normalized to GAPDH. (**C**) HIV-1 virions produced in H9 cells knocked down for endogenous M2 have higher Env levels. Western blots of virions produced in wild type (shCtrl) or M2 depleted (shM2) H9 cells probing with anti-gp120, anti-gp41 and anti-p24 antibodies. Densitometry analysis of gp120 and gp41 band intensity relative to shCtrl and normalized to p24 are shown below (n = 3). (**D**) Endogenous M2 is incorporated into nascent HIV-1 virions. Virus from **C** was used to detect endogenous M2 by western blots probing with anti-M2 and anti-p24 antibodies. **(E)** M2 and MARCH8 (M8) knockdown verification in THP-1 cells depleted for either M2, M8 or both M2 and M8. In the left panel, fold expression changes of human *M2* and *M3* transcripts in shM2 expressing THP-1 cells relative to shCtrl expressing cells and normalized to GAPDH. In the right panel, fold expression changes of human *M2* and *M8* transcripts in shM2, shM8 and shM2/M8 expressing THP-1 cells relative to shCtrl expressing cells normalized to GAPDH. Bottom panels show western blots of endogenous M2 proteins in shCtrl- or shM2, shM8, shM2/M8- expressing THP-1 cells. β-Actin serves as loading control. **(F)** Endogenous M2 has no effect on HIV-1 replication in THP-1 cells. PMA-differentiated THP-1 cells expressing shCtrl or shM2, shM8, both shM2 and shM8 were infected with HIV-1^AD8^ and infected cells were harvested at the indicated time points. HIV-1 *nef* and *env* DNA levels were determined by qPCR and normalized to GAPDH. **(G** and **H)** Only HIV-1 virions produced in THP-1 cells depleted for endogenous M8 have elevated Env levels. PMA-differentiated THP-1 cells expressing shCtrl, shM2, shM8, or shM2/M8 were infected with HIV-1^AD8^, cell lysates and culture supernatants were harvested at 48 hpi. In **G**, culture supernatants were concentrated by ultracentrifugation and p24 normalized purified virions fractions were used for western blot analyses probing for gp120, gp41, p24. The graphs on the right show quantification analysis of virion associated gp120 or gp41 band intensities normalized to p24 band intensities relative to shCtrl conditions. In **H**, p24 normalized purified virions fractions were used to infect TZM-bl cells; luciferase levels were measured 48 hpi, and the percentage (%) of relative infectivity was determined with respect to virus produced in the presence of shCtrl conditions. (**I**) Primary CD4+ T cells were purified from 3 donors, activated, and transduced with lentiviruses expressing either shCtrl or shM2 followed by infection with HIV-1^NL4-3^. Infected cells were harvested at the indicated time points. HIV-1 *nef* and *env* DNA levels were determined by RT-PCR and normalized to GAPDH. (**J**) Primary CD14+ cells were purified from 2 donors, differentiated, and transduced with lentiviruses expressing either shCtrl or shM2, shM8 or both shM2/M8 followed by infection with HIV-1^AD8^. Infected cells were harvested at the indicated time points and analyzed as in **I**. In **A**, **C, D, E** and **G**, representative blots from 3 independent experiments are shown. Graphs in **A**, **B**, **E**, **F**, **G**, **H** represent mean ± SD from 3 independent experiments. Graphs in **I** and **J** represent mean ± SD from 3 and 2 human donors respectively. Statistical analysis in **A**, **B**, **E**, **F**, **I** and **J** performed using unpaired t-test (two-tailed). In **H**, statistical significance was determined using one-sample t-test (two-tailed) when comparisons were performed with shCtrl and unpaired t-test (two-tailed) between non-Ctrl conditions. ns, non-significant; *, *P* ≤ 0.05; **, *P* ≤ 0.01; ***, *P* ≤ 0.001; ****, *P* ≤ 0.0001. (days post infection, dpi).

There is a dearth of information regarding the role of endogenously expressed MARCH proteins during HIV-1 infection. In the case of MARCH2, one previous report showed that it can inhibit infection in Jurkat cells, a T cell stable line [18], but it is well established that stable cell lines do not always reflect what happens in primary cells such as CD4+ T cells and MDMs. Thus, we set to investigate the role of endogenous MARCH2 in both stable cell lines and human primary cells that are naturally infected by HIV-1. Initially, we generated an H9 cell line, in which MARCH2 is stably knocked down, by transducing with lentiviruses expressing either a *MARCH2* specific (shM2) or a control (shCtrl) shRNA. We first verified efficient knockdown of MARCH2 in the lentivirus-transduced H9 cells by RT-PCR, which demonstrated an 80–90% drop of *MARCH2* transcripts (Fig 8A). MARCH3, which shares a high degree of sequence similarity with MARCH2 (64%), served as a control and its expression levels were unaffected (Fig 8A). We further verified MARCH2 knockdown at the protein level in the transduced cell lines by western blot (Fig 8A). MARCH2 is involved in the removal of a number of host immune receptors from the surface of the cell including B7.2 [5]. To ensure that MARCH2 depletion does not affect the surface levels of CD4 and CXCR4, the receptor and co-receptor of HIV-1 in CD4+ T cells, we determined by flow cytometry the levels of these receptors on the surface of MARCH2 expressing or depleted H9 cells and found that they were unaffected (S8A Fig). We then infected wild type (shCtrl) or MARCH2-depleted (shM2) H9 with HIV-1^NL4-3^ and measured HIV-1 DNA levels. We found MARCH2-depleted H9 cells had significantly higher HIV-1 DNA levels compared to MARCH2-expressing H9 cells at all time points (Figs 8B and S8B). To determine if endogenous MARCH2 affects the envelope glycoprotein levels in nascent HIV-1 virions, we purified virus from HIV-1 infected H9 cells expressing or depleted for MARCH2 and performed western blots. We found that virions purified from MARCH2-depleted H9 cells had higher levels of envelope glycoproteins compared to virions produced from MARCH2 expressing H9 cells (Fig 8C). We also observed a band indicative of endogenous MARCH2 in the purified virions obtained from MARCH2-expressing H9 cells which was absent from the virions derived from MARCH2-depleted cells (Fig 8D) suggesting that endogenous MARCH2 is incorporated in nascent viral particles purified from infected H9 cells.

To determine the effect of MARCH2 in THP-1 cells, a monocyte cell line that can be differentiated to macrophages, similar to what we did above with H9 cells, we generated a stable MARCH2 knockdown cell line using a lentivirus transduction system described above and selected for puromycin resistant cells. Additionally, because MARCH8, another member of the MARCH protein family, potently inhibits HIV-1 infection in MDMs [9], we generated THP-1 cells depleted with either a control shRNA, a MARCH8 (shM8) specific shRNA, or shRNAs for both MARCH2 and MARCH8 (shM8/2) followed by selection for geneticin (G418) resistant cells. We first verified efficient knockdown of MARCH2 and MARCH8 by RT-PCR (Fig 8E). Endogenous MARCH2 knockdown in THP-1 cells was further verified at the protein level (Fig 8E). We also confirmed by flow cytometry that the levels of CD4 and CCR5 are unaffected by MARCH2 or MARCH8 depletion in THP-1 cells (S8C Fig). To enhance HIV-1 infection in THP-1 cells, cells were initially treated with SIV Vpx containing virus like particles (VLPs) as previously described [33]. Subsequently, the lentivirus transduced cells were infected with either HIV-1^AD8^ or HIV-1^JR-CSF^, two HIV-1 R5 tropic strains, and at the indicated time points we measured DNA levels. Interestingly, we observed that the presence or absence of MARCH2 in THP-1 cells did not affect HIV-1 DNA levels for both HIV-1^AD8^ (Fig 8F) and HIV-1^JR-CSF^ (S8D Fig). In agreement with previous findings [9], for both HIV-1 strains, upon MARCH8 depletion in THP-1 cells, virus DNA levels were significantly higher than those seen in THP-1 cells treated with shCtrl (Figs 8F and S8D). Moreover, we noticed that HIV-1 DNA levels in cells depleted for both MARCH2 and MARCH8 were similar to those seen in the THP-1 cells depleted only for MARCH8 (Figs 8F and S8D). Therefore, we concluded that while MARCH2 inhibits HIV-1 infection in T cells, MARCH8 is responsible for restricting HIV-1 in macrophages. HIV-1 viral particles purified from infected THP-1 cells depleted for either MARCH2, MARCH8 or MARCH2/MARCH8 were then examined for envelope glycoprotein levels by western blots and densitometry. We observed that HIV-1 virions purified from MARCH2-depleted THP-1 cells had similar envelope glycoprotein content as those purified from THP-1 cells treated with shCtrl (Fig 8G). On the other hand, HIV-1 viral particles purified from MARCH8-depleted or MARCH2/MARCH8-depleted THP-1 cells had higher levels of envelope glycoproteins compared to those purified from shCtrl treated THP-1 cells (Fig 8G). In line with the above findings, when measuring viral particle infectivity of HIV-1 virions produced from the shRNA knockdown THP-1 cells mentioned above, we found that virions isolated from THP-1 cells depleted for MARCH8 were significantly more infectious compared to those originating from wild type THP-1 cells or THP-1 cells depleted for only MARCH2 (Fig 8H).

We then examined the effect of endogenous MARCH2 on HIV-1 infection in CD4+ T cells and MDMs. We isolated primary human CD4+ T cells and MDMs from the buffy coats of human donors (New York Blood Center). Human primary CD4+ T cells were transduced with a lentivirus carrying either a MARCH2 or a control shRNA. We first verified MARCH2 knockdown in the transduced cells by RT-PCR (S8E Fig) and that MARCH2 knockdown does not affect CD4, CXCR4 levels in primary CD4+ T cells (S8F Fig). MARCH2 knockdown and control treated primary CD4+ T cells were then infected with HIV-1^NL4-3^. We found that MARCH2 depleted primary human CD4+ T cells had higher HIV-1 DNA levels compared to MARCH2 expressing (shCtrl) cells (Fig 8I). In the case of MDMs, cells from two anonymous donors were treated with either control shRNA, a MARCH2-specific shRNA followed by puromycin selection. In parallel, MDMs were also treated with another control shRNA, MARCH8-specific shRNA alone or in combination with a MARCH2-specific shRNA followed by selection with geneticin. We initially verified knockdown efficiency of the targeted gene by RT-PCR (S8G Fig) and that MARCH2 and MARCH8 knockdown did not affect CD4 and CCR5 levels in MDMs by flow cytometry (S8H Fig). Subsequently, MDMs were treated with a SIV Vpx containing VLPs and 24 hours later were infected with HIV-1^AD8^. We found that, similar to what we observed in our THP-1 experiments (Fig 8F), MARCH2 depletion had no effect in HIV-1 DNA levels when compared to shRNA Ctrl treated cells (Fig 8J). On the other hand, MDMs depleted for MARCH8 had significantly higher HIV-1 DNA levels when compared to shRNA Ctrl treated cells (Fig 8J). Moreover, MDMs depleted for both MARCH2 and MARCH8 had similar HIV-1 DNA levels as those depleted for only MARCH8 indicating that MARCH2 has no antiviral effect against HIV-1 in MDMs (Fig 8J). In summary, we determined that endogenous MARCH2 restricts HIV-1 infection in a CD4+ T cell specific manner, while MARCH8 inhibits HIV-1 in MDMs.

### MARCH2 inhibits HIV-1 cell-to-cell transmission

**Fig 9:**
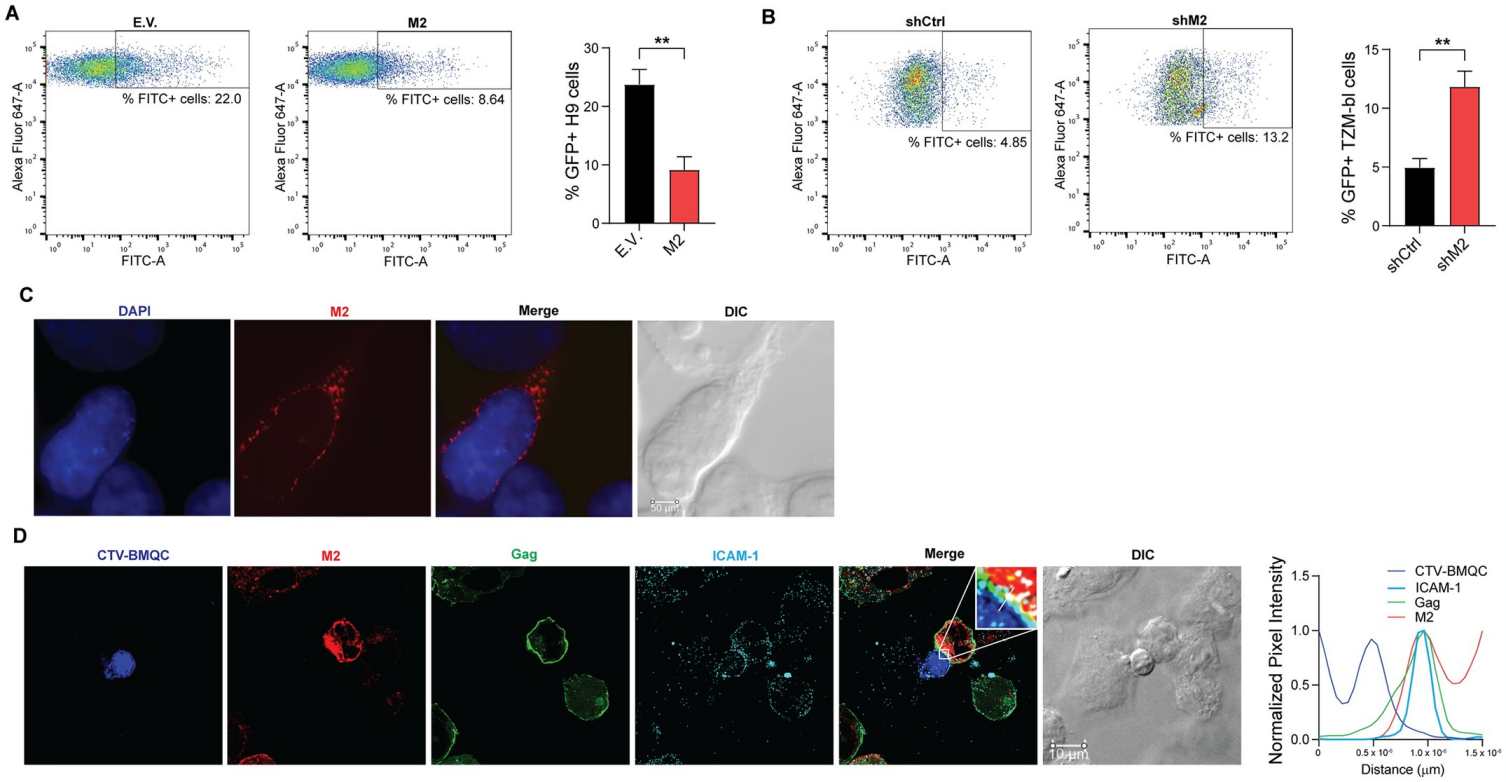
MARCH2 inhibits HIV-1 cell-to-cell transmission and is recruited at virological synapse. **(A)** MARCH2 (M2) overexpression reduces HIV-1 cell-to-cell transfer. CellTrace Far Red (CTFR)-labelled H9 cells were cocultured with 293T donor cells co-transfected with plasmids for HIV Gag-iGFP along with either M2 or empty vector (E.V.). At 16 h of co-culture, cells were harvested and subjected to FACS. **(B)** Endogenous M2 reduces HIV-1 cell-to-cell transfer. CTFR-labelled TZM-bl cells were cocultured with H9 cells stably expressing shRNA-M2 or shRNA-Ctrl and infected with HIV Gag-iGFP. Cells were harvested 24 h post co-culture followed by FACS analysis. **(C)** M2 localizes predominantly in intracellular compartments. AD293 cells transfected with an M2 expressing plasmid were subjected to immunostaining. **(D)** M2 is recruited at the virological synapse during HIV-1 cell-to-cell transmission. CellTracker Voilet (CVT)-BMQC-labelled H9 cells were co-cultured with AD293 cells co-transfected with plasmids expressing HIV Gag-iGFP and M2 followed by immunostaining. Insets represent 55.6 × zoomed images from the indicated boxed regions. The graph at the right shows representative line scanning analyses for co-localization between MARCH2 (M2), HIV-1 Gag (Gag) and ICAM-1. The pixel intensity in each channel is measured along a line drawn on the image (white line in the inset) and relative pixel intensity is plotted versus distance along the line. In **A** and **B**, representative FACS plots and cumulative graphs representing mean ± SD of % GFP+ positive H9 or TZM-bl cells from n = 3 independent experiments are shown. Statistical analysis was performed using unpaired t-test (two-tailed). **, *P* ≤ 0.01. For **C** and **D**, images were acquired from multiple fields from 3 independent experiments and representative deconvolved single Z-section images are shown.

Previous studies have shown that HIV-1 transfer between infected and uninfected T cells can occur via cell-to-cell transmission [34,35]. Because the anti-HIV-1 effect of MARCH2 is T cell specific (Fig 8B and 8I), we examined the effect of this factor on cell-to-cell transmission. For our assays, we used HIV Gag-iGFP, because the GFP insertion between the matrix (MA) and capsid (CA) of Gag attenuates multi-round replication [34,36]. Initially, we used 293T cells co-transfected with HIV Gag-iGFP along with either MARCH2 or E.V. as donors. We then verified that the transfected 293T cells showed similar levels of GFP+ populations and MARCH2 was expressed (S9A and S9B Fig). 293T cells expressing HIV Gag-iGFP were then co-cultured with CellTrace Far Red (CTFR)-labelled H9 cells followed by flow cytometry. We noticed that the number of GFP+ target H9 cells was reduced when donor 293T cells expressed MARCH2 (Fig 9A). As this assay was performed upon overexpression of MARCH2, we investigated the effect of endogenous MARCH2 on HIV-1 cell-to-cell transmission. We used MARCH2-expressing or -depleted H9 cells and infected them with VSV-G pseudotyped HIV Gag-iGFP virus. Upon verification that >90% of H9 cells were GFP+ (S9C Fig), we co-cultured these cells with CTFR-labelled TZM-bl target cells. The number of GFP+ TZM-bl cells was increased when they were co-cultured with H9 cells depleted of MARCH2 compared to those co-cultured with MARCH2 expressing H9 cells (Fig 9B). Hence, we conclude that endogenous MARCH2 inhibits HIV-1 cell-to-cell transmission.

In MARCH2-transfected cells, MARCH2 was detected as punctate structures predominantly in intracellular compartments [5,23] (Fig 9C). Thus, we asked whether MARCH2 is recruited at the sites of virological synapses during cell-to-cell transmission of HIV-1. To address this, we co-transfected AD293 cells with an expression plasmid for HIV Gag-iGFP in the presence or absence of MARCH2. Transfected donor cells were co-cultured with H9 cells loaded with a violet fluorescent dye (CTV-BMQC) followed by confocal immunofluorescence microscopy. Consistent with previous studies [34,37–39], we observed HIV-1 Gag recruitment at the contact zone between donor and target cells (Fig 9D), a hallmark of virological synapse formation. Cells were also stained for a cellular marker of virological synapses, ICAM-1 [40,41]. Confocal analyses showed that MARCH2 was enriched at the cell-to-cell contact zone (Fig 9D). Altogether, these findings show that MARCH2 accumulates at the virological synapses and can inhibit cell-to-cell transmission.

## Discussion

MARCH proteins have emerged as an important family of host factors that inhibit HIV-1 infection by preventing the incorporation of viral envelope glycoproteins into nascent virions [9–11,15]. Nevertheless, many questions remain regarding the mechanism they utilize to block HIV-1 infection [9,10,15,18]. In this study, we examine in depth the antiviral effect of MARCH2 on HIV-1 infection. MARCH2 is found ubiquitously in the host and localizes to various cellular organelles including the plasma membrane and Golgi [23,42]. Many host receptors have been identified as targets of MARCH2 including β2 adrenergic receptor and cystic fibrosis transmembrane conductance regulator (CFTR) [43,44]. In contrast to previous findings [18], *MARCH2* RNA levels in all cell types we tested (H9, THP-1, primary CD4+ T cells and MDMs) were unaffected by HIV-1 infection and IFN-β treatment. We speculate that the discrepancy between our findings and previous results regarding the transcriptional induction of *MARCH2* may be due to technical differences in treatment. Nevertheless, in support of our data, there has been no up to date study that has reported *MARCH2* as an interferon stimulated gene (ISG) or an HIV-1 inducible gene.

Unlike MARCH 1 and 8, for which their antiviral effect is conserved in both murine and human orthologs, only human MARCH2 blocks HIV-1 infection, mouse MARCH2 has no antiviral effect [10]. Interestingly the human and murine orthologs of MARCH2 differ in 10 amino acids clustered in the N- and C- terminal tails of the protein (Fig 2A). In this report, we showed that amino acid Gly61 found in the N-terminal tail of human MARCH2, but absent in mouse MARCH2, is critical for its anti-HIV-1 function. The importance of a single amino acid in altering the antiviral function of host factors has also been demonstrated in the case of TRIM5α. While human TRIM5α has a very modest effect on HIV-1 replication, rhesus monkey TRIM5α potently restricts HIV-1 infection. Alteration of arginine 332 of human TRIM5α to proline, the residue found in rhesus monkey TRIM5α, renders human TRIM5α potently antiviral towards HIV-1 [45,46]. Our findings further support the notion that small changes in the amino acid sequence of proteins during evolution can have drastic effects in their antiviral phenotype. It is possible that Gly61 facilitates the interaction of MARCH2 with a host factor that affects the ability of MARCH2 to inhibit HIV-1, something we will investigate in the future.

MARCH2 is a transmembrane protein with two TM domains, a RING-CH domain [3] and a C-terminal PDZ-binding domain critical for protein-protein interactions [24,47]. In this study, we show that only the RING-CH and the second TM domains are critical for its antiviral function. Interestingly, the PDZ motif that is important for MARCH2-mediated protein-protein interactions [47], did not affect MARCH2 inhibition of HIV-1. In addition, we found that MARCH2 interacts with the HIV-1 envelope glycoproteins via TM-TM interactions. Transmembrane domain-mediated interactions of host restriction factors with viral proteins may have important benefits for the host due to the fact that transmembrane sequences, like that of gp41, can be quite stable and highly conserved [48]. Mutations in TM domains may result in structural changes affecting membrane localization of a protein thereby impacting its function [49]. In addition, the importance of the MARCH2 TM domains on HIV-1 restriction is further evident when we investigated the MARCH2 isoforms for antiviral function. Our studies showed that only the long isoform of MARCH2 (*March2-001*), which contains both TM domains, can restrict HIV-1, whereas the short isoform does not. The fact that not all MARCH2 isoforms have antiviral function is similar to what has been seen with SERINC5 and Mx2, two other known host restriction factors [50,51]. Nevertheless, the function of the short MARCH2 isoform remains to be elucidated.

We also demonstrate that MARCH2 is incorporated inside the viral particles in a TM domain dependent manner. Future studies will focus on determining the specific residues in HIV-1 gp41 and MARCH2 that are responsible for this TM-TM interaction. MARCH2 incorporation in nascent virions was unexpected, as MARCH2 localizes in intracellular membrane structures. However, we observed that in the presence of HIV-1, MARCH2 was detected at the plasma membrane. The mechanism of MARCH2 translocation to the cell surface upon infection is unknown. The virion incorporation of MARCH2 was further validated with virus purified from cells expressing endogenous levels of MARCH2. Due to the limited space inside the viral particle, it is well established that host factors that are incorporated inside HIV-1 virions many times can have important antiviral function as is the case of SERINC5 and APOBEC3G [52–54]. In this report, we demonstrate a novel mode of restriction by MARCH2. We found that MARCH2 incorporated in viral particles reduces particle infectivity in a RING-CH independent manner by interfering with virus-cell membrane fusion. Thus, MARCH2 utilizes two mechanisms to block HIV-1 infection, (1) it blocks envelope incorporation in producer cells and (2) by getting incorporated into HIV-1 virions, reduces viral particle infectivity. Whether the effect of MARCH2 on particle infectivity extends to the other MARCH members remains to be elucidated. Another family of host factors, the interferon-induced transmembrane (IFITM) proteins, also restricts HIV-1 by preventing both envelope glycoprotein incorporation into nascent virions and by incorporation into viral particles thereby interfering with HIV-1 virion infectivity [55–57]. In summary, our findings with MARCH2 show that this protein family utilizes multiple strategies to counteract viral infections.

MARCH8 inhibits HIV-1 replication in MDMs only, as it is expressed at very low levels in CD4+ T cells [9]. As multiple MARCH protein members restrict HIV-1 infection, we wondered if restriction is cell type-dependent. We found that MARCH2, unlike MARCH8 [9], is expressed at high levels in both CD4+ T cells and in MDMs. However, the antiviral effect of MARCH2 on HIV-1 replication is only seen in CD4+ T cells and not in MDMs. Moreover, we show that endogenous MARCH2 interacts with the HIV-1 gp41, is incorporated in nascent virions and decreases envelope glycoprotein levels in newly synthesized viral particles. On the other hand, MARCH8 inhibits HIV-1 infection in THP-1 cells and MDMs, which is in agreement with previous findings [9]. The fact that MARCH8 and MARCH2 inhibit HIV-1 infection in different cell types (MDMs vs. CD4+ T cells respectively) may be a reason behind the fact that multiple members of this family of host factors inhibit HIV-1 infection utilizing the same mechanism. A cell type restrictive effect by a host factor has also been previously reported with other host factors [e.g., Mannose receptor [58,59]]. The reason why MARCH2, while expressed highly in MDMs, has no antiviral effect in these cells may be due to the absence of a cellular factor that MARCH2 may need to exert its antiviral effect and may be highly expressed in primary CD4+ T cells. Additional studies are needed to elucidate the cell type specific effect of MARCH2. Efficient HIV-1 spread from infected T cells to uninfected cells occurs via cell-to-cell transmission and not by cell-free infection [60]. Here we show that cell-to-cell transmission of HIV-1 is decreased in the presence of MARCH2 and MARCH2 localizes at the virological synapse. As CD4 and CXCR4 are not affected, we do not think MARCH2 is disrupting the cellular receptors found at the virological synapse. In conclusion, our findings reveal new aspects of the antiviral function of the MARCH protein family and demonstrate their importance in host defense during HIV-1 infection.

## Materials and methods

### Ethics statement

Buffy coats isolated from anonymous healthy donors by apheresis were obtained from the New York Blood Center, Long Island City, NY. The use of de-identified human blood from anonymous donors is considered non-human subject research in accordance to federal regulations and thus does not require formal IRB review.

### Cell culture

293T cells (ATCC), TZM-bl cells (NIH HIV Reagent Program, Division of AIDS, NIAID, NIH: ARP-8129), AD293 cells (Agilent), and NIH 3T3 cells were cultured in Dulbecco’s Modified Eagle Media (DMEM; Gibco) with 10% (vol/vol) fetal bovine serum (FBS; Sigma), 6 mM L-glutamine (Gibco), and 100 mg/ml penicillin and streptomycin (P/S; Gibco) (complete DMEM). U373-MAGI-CXCR4 cells (NIH HIV Reagent Program, Division of AIDS, NIAID, NIH: ARP-3596) were cultured in complete DMEM (10% FBS, 100 mg/ml P/S) with 0.2 mg/ml G418 (Gibco), 0.1 mg/ml hygromycin B (Invitrogen), and 1.0 μg/ml puromycin (Research Products International). H9 cells (ATCC) were cultured in RPMI 1640 media (Gibco) with 10% FBS and 100 mg/ml P/S (complete RPMI). THP-1 cells (ATCC) were cultured in complete RPMI media supplemented with 0.05 mM β- mercaptoethanol. For THP-1 differentiation, cells were treated with 50 nM of Phorbol 12-myristate 13- acetate (PMA; MedChemExpress) for 2 days and maintained for an additional 24 h without PMA prior to infection.

PBMCs were isolated from blood of healthy donors by density gradient centrifugation on Ficoll-Paque Plus (GE Healthcare). CD4+ T cells and monocytes were isolated from PBMCs using EasySep Human CD4+ T Cells Isolation Kit (Stemcell Technologies) and EasySep Human CD14 Positive Selection Kit (Stemcell Technologies) respectively according to manufacturer’s guidelines. Purified CD4+ T cells were activated by treatment with 2.5 μg/ml of anti-human CD3 antibody (Thermo Fischer Scientific) and 1.25 μg/ml of anti-human CD28 antibody (Thermo Fischer Scientific) and were maintained in complete RPMI media supplemented with 10 U/ml of recombinant human interleukin-2 (IL-2) (Gibco) for 5 days. Purified CD14+ monocytes were differentiated to monocyte-derived macrophages (MDMs) in complete RPMI media supplemented with 50 ng/ml of recombinant human granulocyte-macrophage colony-stimulating factor (GM-CSF) (Stemcell Technologies) for 7 days.

### Plasmids

The MLV molecular clone (pLRB302), HIV-1 molecular clones: pNL4-3, HIV Gag-iGFP and JR-CSF (pYK-JRCSF) used in this paper have been previously described [36,61–63]. The pFB-*luc* construct and VSV-G expression vector have been previously described [10,64]. The following reagents were obtained from the NIH AIDS Reagent program, Division of AIDS, NIAID, NIH: HIV-1 NL4-3 Infectious Molecular Clone (pNL4-3), ARP-2852, contributed by Dr. M. Martin; HIV Gag-iGFP, ARP-12457, contributed by Dr. Benjamin Chen; (HIV-1), JR-CSF Infectious Molecular Clone (pYK-JRCSF), ARP-2708, contributed by Dr. S. Y. Chen and Dr. Yoshio Koyanagi; HIV-1 NL4-3 ΔEnv Vpr Luciferase Reporter Vector (pNL4-3.Luc.R-E-), ARP-3418, contributed by Dr. Nathaniel Landau, HIV-1 YU-2 Vpr β-lactamase Expression Vector (pMM310), APR-11444, contributed by Dr. Michael Miller (Merck Research Laboratories), and HIV-1 NL4-3 AD8 Infectious Molecular Clone (AD8), ARP-11346, contributed by Dr. Eric O. Freed.

Human MARCH2 construct has been previous described [18]. Cloning strategies for human MARCH2 (hMARCH2; represents the longer isoform 1 unless stated otherwise), human MARCH4 and mouse MARCH2 (mMARCH2) into pcDNA3.1/Myc-His A (Invitrogen) and pBJ5 vector have been previously described [10]. Human MARCH2 isoform 2 was identified from https://www.uniprot.org/uniprotkb/Q9P0N8/entry#Q9P0N8-1/2 and was acquired from GenScript, (clone ID: OHu31844, Accession Version: NM_001005416.2) followed by cloning into pcDNA3.1/Myc-His A vector using the same primers for the human MARCH2 isoform 1. MARCH2 chimeras containing N-terminal swaps (pBJ5 mMARCH2^N^–hMARCH2^C^ or pBJ5 hMARCH2^N^–mMARCH2^C^) were generated by using site-directed mutagenesis (SDM) to eliminate an ApaI cut site (nucleotide position 679) in hMARCH2 construct using a pair of hMARCH2ApaIuncut primers listed in S2 Table, thus ApaI can only digest hMARCH2 at one site (nucleotide position 489). This modified hMARCH2 and mMARCH2 were then cut with XhoI-ApaI and ApaI-NotI to isolate the N-terminal and C-terminal fragments followed by reciprocal ligations to generate the indicated chimeras. MARCH2 mutant constructs containing point mutations and deletions used in this study were generated by SDM using Q5 polymerase (New England Biolabs) and primers listed in S2 Table. MARCH2 chimeras containing the TM domains from either human MARCH4 or human transferrin receptor (TR) were generated using the NEBuilder HiFi DNA assembly kit (New England Biolabs) and the primers listed in S2 Table. TM domain of TR was PCR amplified from 293T cDNA as template. For construction of pcDNA3.1 NL4-3 Env, we initially digested the pNL4-3 plasmid with EcoRI and XhoI and subcloned this fragment into pcDNA3.1/Myc-His A (Invitrogen). To generate pcDNA3.1 NL4-3 TM^TR^ Env, we replaced NL4-3 Env TM domain with that of TR using the NEBuilder HiFi DNA assembly kit and the primers listed in S2 Table. Plasmid eGFP-N1 (Clontech) was digested with HindIII and NotI and cloned into pcDNA3.1/Myc-His A to generate pcDNA3.1 eGFP-N1. All constructs generated were confirmed by DNA sequencing. pSIV-D3psi/delta env/delta Vif/delta Vpr (132928), Ubiquitin-HA (18712) and NEMO-FLAG (11970) plasmids were obtained from Addgene.

### Virus preparation

Virus and SIV Vpx containing VLP stocks (HIV-1 NL4-3, HIV-1 AD8, HIV-1 JR-CSF, HIV Gag-iGFP, and pSIV-D3psi/delta env/delta Vif/delta Vpr) were prepared by transfecting 293T cells (3 × 10^6^ cells) seeded in 10-cm-diameter cell culture dishes with 10 μg of plasmid DNA mentioned above. When pseudotyping with VSV-G, 1 μg of VSV-G expression vector was included during co-transfection. Culture media was removed and replenished 24 h after transfection. All culture supernatants were harvested 48 h after transfection, centrifuged at 714 × g at 4°C to eliminate cellular debris, filtered through 0.45 μm filter and treated with 10 U/ml DNase I (Roche) for 30 min at 37°C. Aliquots of filtered culture supernatants were stored at −80°C for future experiments. HIV-1 p24 levels were determined by using a HIV-1 p24 ELISA kit (Xpress Biotech International).

### HIV-1 infection, interferon treatment and human *MARCH2* expression analysis

THP-1 cells were treated with 50 nM of phorbol 12-myristate 13-acetate (PMA) (MedChemExpress) for 2 days. PMA-differentiated THP-1 cells and H9 cells were seeded at a density of 5 × 10^4^/well in a 96-well plate and treated with or without 500 U/ml of human Interferon Beta 1β (PBL Assay Science). Activated CD4+ T cells (1 × 10^5^/ well) and differentiated MDMs (5 × 10^4^/well) seeded in a 24-well plate were treated with or without 500 U/ml of human IFN-β (PBL Assay Science). Cells were harvested at 4, 8, 16, and 24 h post treatment and RNA was isolated using RNeasy Mini kit (Qiagen). cDNA was synthesized using the SuperScript III First Strand Synthesis kit (Invitrogen) per manufacturer’s recommendation. RT-PCR was performed using the PowerUp SYBR Green PCR master mix kit (Applied Biosystems) in a CFX384 Touch Real-Time PCR detection system (Bio-Rad). The following primers were used; *human MARCH2*: 5’-GCTGTCTGGAGAAGTGGCTT-3’/5’-CTTCAGCCACTCTGTGAGGG-3’, *GAPDH* 5′-AACGGGAAGCTTGTCATCAATGGAAA-3′/5′-GCATCAGCAGAGGGGGCAGAG-3′ and *Interferon-stimulated gene 15* (*ISG15*): 5′- GATCACCCAGAAGATCGGCG-3’/5’- GGATGCTCAGAGGTTCGTCG-3′.

PMA-differentiated THP-1 and H9 cells seeded at a density of 5 × 10^4^ cells per well in a 96-well plate were infected with a 5 MOI of HIV-1 (HIV-1^JR-CSF^ and HIV-1^NL4-3^) respectively by spinoculation as previously described [65]. Cells were harvested at 4, 8, 16, and 24 h post infection. RNA isolation, cDNA synthesis and RT-PCR were performed as mentioned above. To quantify total HIV-1 DNA in the infected cells, DNA was extracted using a DNeasy blood and tissue kit (Qiagen) and RT-PCR was performed as mentioned above using the following primers: NL4-3 *Nef*: 5’-CAAGTGGTCAAAAAGTAGTGTGATT-3’/ 5’-ATACTGCTCCCACCCCATC-3’ and JR-CSF *Nef*: 5’-CACAAGGCTACTTCCCTGATT-3’/5’-CTCCTTCATTGGCCTCTTCTAC-3’. The relative levels of amplification were quantified for each sample from standard curves generated using known quantities of DNA standard templates and normalized to GAPDH levels.

### Transfection and immunoblotting

All transfections were performed on 293T cells (seeded at a density of 0.5 × 10^6^ cells/well of a 6- well plate one day prior) using Lipofectamine 3000 transfection reagent (Thermo Fisher Scientific) according to the manufacturer’s recommendation unless stated otherwise. 293T cells were co-transfected with plasmids for MLV (pLRB302, 3 μg) or HIV-1 NL4-3 (2 μg) along with either 3 μg of pBJ5 MARCHs (mMARCH2; hMARCH2; mMARCH2^N^–hMARCH2^C^; hMARCH2^N^–mMARCH2^C^; mMARCH2 mutants: S18G, Q58P, C61G, N75C; hMARCH2 mutants: G18S, P58Q, G61C, C75N) or empty vector. To determine the effect of human MARCH2 polymorphisms, we co-transfected 293T cells with pNL4-3.Luc.R-E- (2.5 μg), pcDNA3.1 NL4-3 Env (1.25 μg) and either pBJ5 MARCH2 plasmids (1, 2, and 4 μg) or empty vector. Culture media was removed and replenished 24 h after transfection. Virus containing culture supernatants and cells were harvested 48 h after transfection. Culture supernatants were processed as described before (see *Virus preparation* section) and used for subsequent infection experiments. Cells were lysed in 1× RIPA buffer (150 mM NaCl, 1% NP-40, 0.5% sodium deoxycholate, 0.1% SDS, 25 mM Tris, pH 7.4, with Halt phosphatase and protease inhibitors). Cell lysates and virus pellets were mixed with 1 × sample loading buffer, boiled at 100°C for 10 minutes and resolved on either 8%, 10%, 12%, or 15% sodium dodecyl sulfate-polyacrylamide gels (SDS-PAGE) as needed. Blots were probed using the following antibodies: goat anti-MLV gp70 [66], rat anti-MLV p15E (clone 42/114; Kerafast), rat anti-MLV p30 (R187, ATCC CRL-1912), rabbit anti-Myc (Cell Signaling Technology), mouse anti-Myc (Cell Signaling Technology), sheep anti-HIV-1 gp120 (NIH HIV Reagent program, ARP-288), mouse anti-HIV gp41 (Chessie8) (NIH HIV Reagent program, ARP-526), human anti-HIV gp41 clone 2F5 (NIH HIV Reagent program, ARP-1475), mouse anti-HIV p24 (NIH HIV Reagent program, ARP-4121), rabbit anti-MARCH2 (Invitrogen, PA5-30620), rabbit anti-GAPDH 14C10 (Cell Signaling Technology), mouse anti-HA (Invitrogen, 26183), rabbit anti-FLAG (Cell Signaling Technology) and monoclonal anti-β-actin (Sigma-Aldrich). Horseradish peroxidase (HRP)-conjugated anti-rabbit IgG (Cell Signaling Technology), HRP-conjugated anti-Sheep IgG (Thermo Fischer Scientific), HRP-conjugated anti-rat IgG (Cell Signaling Technology), HRP-conjugated anti-human IgG (Sigma-Aldrich), HRP-conjugated anti-mouse IgG (EMD Millipore), HRP-conjugated anti-goat IgG (Sigma-Aldrich), and light chain specific HRP-conjugated anti-mouse IgG (Cell Signaling Technology) were used for detection using the enhanced chemiluminescence detection kits Clarity and Clarity Max ECL (Bio-Rad).

To determine the importance of the human MARCH2 domains on its antiviral function, we co-transfected 293T cells with a plasmid for HIV-1 NL4-3 (1.2 μg) along with pcDNA3.1 hMARCHs (250 ng of hMARCH2, hMARCH2 Δ2–30, hMARCH2 Δ31–56, hMARCH2 PDZ mutant; 70 ng of hMARCH2 W97A and hMARCH2 C64/67S RING-CH mutants; 200 ng of hMARCH2 TM chimeras). For experiments with human MARCH2 isoforms, we co-transfected 293T cells with pNL4-3.Luc.R-E- (2.5 μg), pcDNA3.1 NL4-3 Env (1.25 μg) along with 700 ng of either pcDNA3.1 hMARCH2 isoform 1, pcDNA3.1 hMARCH2 isoform 2 or empty vector. In our experiments with HIV-1 envelope TM domain swap, we co-transfected 293T cells using 3 μg of either pcDNA3.1 NL4-3 Env or NL4-3 TM^TR^ Env along with either 2 μg of pBJ5 hMARCH2 or empty vector. For experiments with increasing concentrations of hMARCH2 RING-CH mutant, 293T cells were co-transfected with pNL4-3.Luc.R-E- (2.5 μg), pcDNA3.1 NL4-3 Env (1.25 μg) along with varying concentrations of pcDNA3.1 hMARCH2 RING-CH mutant (25, 50, and 100 ng) or empty vector. For experiments with increasing concentrations of hMARCH2 TM2^TR^ mutant, 293T cells were co-transfected with pNL4-3.Luc.R-E- (2.5 μg), pcDNA3.1 NL4-3 Env (1.25 μg) along with either pcDNA3.1 hMARCH2 WT (10 ng) and varying concentrations of pcDNA3.1 hMARCH2 TM2^TR^ mutant (25, 50, 100 and 200 ng) or empty vector. For experiments determining the effect of virion- incorporated MARCH2 on HIV-1 infectivity, we co-transfected 293T cells with pNL4-3 (2 μg) along with either pBJ5 hMARCH2 (1 or 2 μg) or empty vector. For experiments with virion- incorporated hMARCH2 RING-CH mutant, we co-transfected 293T cells with pNL4-3.Luc.R-E- (2.5 μg), pcDNA3.1 NL4-3 Env (1.25 μg) along with either pcDNA3.1 hMARCH2 WT (700 ng), pcDNA3.1 hMARCH2 RING-CH mutant (100 ng) or empty vector. To determine if mouse MARCH2 can be incorporated in virions, we co-transfected 293T cells with pNL4-3.Luc.R-E- (2.5 μg), pcDNA3.1 NL4-3 Env (1.25 μg) along with either pcDNA3.1 mMARCH2 WT (250 ng), pcDNA3.1 mMARCH2 RING-CH mutant (120 ng) or empty vector. Culture supernatants and cell lysates were harvested 48 h post transfection and processed as described above.

For the detection of endogenous human MARCH2, H9 cells were lysed in 1× RIPA buffer, THP1 cells were lysed in DM lysis buffer (0.5% [wt/vol] n-decyl-β-D-maltopyranoside, 20 mM Tris-HCl, pH 7.5, 10% [vol/ vol] glycerol, 1× Halt protease inhibitor cocktail, Benzonase [25 U/ml]) and proteins were resolved in a 15% SDS-PAGE. Band intensities were determined by densitometry using ImageJ software (National Institutes of Health; https://imagej.nih.gov/ij/).

### Membrane fractionation assay

To ensure that MARCH2 mutants localized to cellular membranes, we used the Mem-PER Plus membrane extraction kit (Thermo Fisher Scientific) per manufacturer’s recommendation. Transfection conditions for the expression of wild type MARCH2 and the various MARCH2 mutants in 293T cells has been previously described (see *Transfection and Immunoblotting* section). Membrane fraction purity was verified by western blots probing for rabbit anti-GAPDH.

### Luciferase assay

293T cells were co-transfected with an MLV molecular clone (pLRB302 3 μg), pFB-luc (1 μg), and 4 μg of either pBJ5 hMARCH2, mMARCH2 or pBJ5 mMARCH2 carrying the human amino acids at the indicated positions (S18G, Q58P, C61G, and N75C) or empty vector. Culture supernatants were harvested 48 h post transfection and processed as described above (see *Virus preparation* section). NIH 3T3 cells were seeded in 12-well plates (0.9 × 10^5^ cells/well). Cells were infected the next day with culture supernatants and lysed 48 hpi followed by measuring luminescence using the Steady-Glo luciferase assay system (Promega) per the manufacturer’s recommendation and a Biostack4 (BioTek) luminometer. Luminescence values were normalized to MLV p30 levels.

We generated pseudoviruses for experiments with human MARCH2 amino acids substitutions from that of mouse MARCH2 or mouse MARCH2 C61G by co-transfecting 293T cells with pNL4-3.Luc.R-E- (2.5 μg), pcDNA3.1 NL4-3 Env (1.25 μg) along with 4 μg of either pBJ5 mMARCH2, hMARCH2, hMARCH2 variants with the mouse MARCH2 amino acids at the indicated positions (G18S, P58Q, G61C, and C75N), mMARCH2 C61G or empty vector. Transfection conditions for pseudovirus production with human MARCH2 polymorphic variants, isoforms, and RING-CH mutant (pcDNA3.1 eGFP-N1 was used as E.V.) and increasing concentrations of the MARCH2 TM2^TR^ mutant have been described above (see *Transfection and Immunoblotting section*). Culture supernatants were processed as described above and used to infect U373-MAGI-CXCR4 cells (0.5 × 10^5^ cells/well in a 24-well plate). Cells were lysed 48 hpi followed by measuring luminescence as described above. Luminescence values were normalized to HIV-1 p24 levels.

Transfection conditions for determining the infectivity of HIV-1 containing human MARCH2 have been described above (see *Transfection and Immunoblotting section*). Culture supernatants were harvested as described above and HIV-1 gp120 levels were determined using a HIV-1 gp120 ELISA kit (SinoBiological). HIV-1 gp120 normalized culture supernatants were used to infect TZM-bl cells (0.2 × 10^6^ cells/well in a 12-well plate). Six hpi, cells were maintained in the presence of 500 nM fusion inhibitor enfuvirtide (T20; NIH HIV Reagent program, HRP-12732). Cells were lysed 48 hpi followed and luminescence was measured as described above. Particle infectivity was expressed as luciferase values normalized to p24 levels. Infectivity of HIV-1^AD8^ virions produced in THP-1 wild type cells or cells depleted for either MARCH2, MARCH8, or for both MARCH factors were determined by infecting TZM-bl cells as mentioned above.

MLV p30 or HIV p24 levels on culture supernatants was determined by immunoblotting (see *Transfection and Immunoblotting* section) followed by densitometry using ImageJ software (National Institutes of Health; https://imagej.nih.gov/ij/).

### Coimmunoprecipitations (coIPs)

For our pulldown experiments for NEMO, 293T cells were co-transfected with 0.5 μg of Ubiquitin-HA, 0.5 μg of NEMO-FLAG and pBJ5 hMARCH2 (MARCH2 WT: 3 μg, MARCH2 W97A: 0.75 μg, and MARCH2 G61C: 0.75 μg) plasmids. Cells were harvested 48 h post-transfection, washed with cold 1× PBS and lysed in 350 μl of 1× RIPA buffer. Following centrifugation, clarified lysates (500 μg) were used for immunoprecipitation. CoIPs were performed using the Dynabeads protein A immunoprecipitation kit (Thermo Fisher Scientific) per manufacturer’s instructions with some modifications. Briefly, 50 μl of beads were incubated with anti-FLAG antibody (Cell Signaling Technology) at 1:100 dilution for 20 minutes at RT. Beads were then washed and incubated with cell lysates at 4°C overnight. The following day, beads were washed, eluted, and the eluted fractions were subjected to immunoblotting. Antibodies used to probe western blots are described above (see *Transfection and Immunoblotting* section).

For our coIP experiments examining endogenous human MARCH2, we pelleted 10 × 10^6^ H9 cells and infected with an 0.1 MOI of HIV-1 NL4-3 in a total volume of 2 ml complete RPMI media containing 2 μg of polybrene for 2 h at 37°C. Cells were washed once to remove viral inoculum and maintained in 20 ml of complete RPMI media. Cells were harvested at 5 dpi, washed twice with cold 1× PBS and lysed in 1 ml of 1× RIPA. Following centrifugation, clarified lysates (1000 μg) were used for immunoprecipitation. CoIPs were performed using the Dynabeads protein A immunoprecipitation kit (Thermo Fisher Scientific) per manufacturer’s instructions with some modifications. Briefly, cell lysates were incubated with mouse anti-HIV-1 gp41 (Chessie 8) (NIH HIV Reagent program, ARP-526) at a 1: 200 dilution overnight at 4°C. Next day, 50 μl protein A Dynabeads was added and incubated for 1 h at RT. Dynabeads were then washed and eluted. The eluted fractions were subjected to western blot analysis. Antibodies used to probe western blots are described above (see *Transfection and Immunoblotting* section).

For our coIP experiments examining the role of the human MARCH2 TM domains, we co-transfected 293T cells with 1.2 μg of HIV-1 molecular clone (pNL4-3) along with pcDNA3.1 hMARCH2 plasmids (100 ng of hMARCH2, hMARCH2 TM2^TR^; 25 ng of hMARCH2 TM1^M4^) or empty vector. At 48 h post-transfection, cells were lysed in IP lysis buffer (25 mM Tris pH 7.4, 150 mM NaCl, 2 mM MgCl_2_, 1% Triton X-100, 5% Glycerol, Benzonase [25 U/ml], 1× Halt protease inhibitor cocktail). Following centrifugation, clarified lysates (1000 μg) were incubated with 25 μl of Protein A Dynabeads for 6 h at 4°C. Precleared lysates were incubated with mouse anti-HIV-1 gp41 (Chessie 8) as described above. Next day, 30 μl protein A Dynabeads was added and incubated for 1 h at RT followed by 2 h incubation at 4°C. Finally, Dynabeads were then processed as mentioned above.

For coIPs examining the role of the HIV-1 TM domain, we co-transfected 293T cells with 4 μg of pcDNA3.1 NL4-3 Env, 3 μg of pcDNA3.1 NL4-3 TM^TR^ Env, and 50 ng of pcDNA3.1 hMARCH2 or empty vector. Cells were harvested, precleared, and incubated with mouse anti-HIV-1 gp41 (Chessie 8) as described above. Next day, 30 μl protein A Dynabeads was added and incubated for 2 h at RT followed by immunoblot analysis as described above.

### MARCH2 structure prediction with AlphaFold

Predicted structures of human MARCH2 (UniProt ID: Q9P0N8) and human MARCH2 G61C were modeled using AlphaFold [67]. The predicted structure files (.pdb) of the two proteins with the highest score (0.1131523598666395 for human MARCH2, 0.11225345598577166 for human MARCH2 G61C) are shown in S2D Fig. Models were visualized using iCn3D [68].

### OptiPrep gradient separation

293T cells (3 × 10^6^) seeded in 10-cm-diameter cell culture dishes were co-transfected with plasmids for HIV-1 NL4-3 (10 μg) and hMARCH2 constructs (1 μg). Culture media was removed and replenished 24 h after transfection. Culture supernatants were harvested 48 h after transfection, filtered through 0.45 μm filter and pelleted through a 30% sucrose cushion and resuspended in 200 μl of 1× PBS as previously described [69]. A 5% to 20% OptiPrep (60% [wt/vol] iodixanol; Stemcell Technologies) gradient was prepared in PBS using a GRADIENT MASTER 108 (Biocomp) instrument. Concentrated virions were layered onto the top of the gradient and centrifuged at 250,000 × g for 90 minutes at 4°C in a SW41Ti swinging bucket rotor. Fifteen gradient fractions (~ 750 μl each) were collected from the top of gradient using a Brandel tube piercer along with Fluorinert FC-40 (Sigma-Aldrich) [70]. Proteins were recovered from the fractions by TCA precipitation and analyzed by immunoblotting (see *Transfection and Immunoblotting* section).

### BlaM-Vpr fusion assay

To generate BlaM-Vpr containing HIV-1 viruses in the presence or absence of human MARCH2, transfections were performed as described above (see *Virus preparation* section) along with plasmids for HIV-1 YU2 Vpr β-lactamase expression (2.5 μg), pcDNA3.1 hMARCH2 RING-CH mutant (2 μg) or empty vector. Culture supernatants were harvested 48 h post transfection, pelleted by ultracentrifugation as described above (see *OptiPrep gradient separation* section) and resuspended in 600 μl of complete DMEM media. Virion fusion assays were performed as previously described [31,71]. Briefly, 1 × 10^6^ H9 cells seeded per well of a 12- well culture plate were infected with 250 ng of p24 equivalent virions by spinoculation for 2 h at 257 × g at RT in the presence or absence of 500 nM fusion inhibitor enfuvirtide (T20; NIH HIV Reagent program, HRP-12732). Cells were shifted to 37°C for 1 h, then washed and stained with 6× CCF2-AM substrate loading solution (Invitrogen) for 1 h at RT. Cells were washed and maintained in development media (CO_2_-independent media [Gibco] with 2.5 mM probenecid [Sigma-Aldrich]) for 16 h at RT in dark. Subsequently, cells were washed, fixed and processed via BD FACSCelesta followed by data analysis using FlowJo version 10.8.0.

### shRNA knockdown of human MARCH2 and MARCH8 in cell lines and primary cells followed by HIV-1 infection

The following oligos were used to construct pLKO.1 (Addgene; 10878) expressing shRNA to hMARCH2 (shRNA-M2): FW sh-hM2-2 5’-CCGGGGAGAAAGACCAACCAGAAAGCTCGAGCTTTCTGGTTGGTCTTTCTCCTTTTT-3’/RE sh-hM2-2 5’-AATTCAAAAAGGAGAAAGACCAACCAGAAAGCTCGAGCTTTCTGGTTGGTCTTTCTCC-3’ for puromycin selection. The following oligos were used to construct pLKO.1 (Addgene; 13425) expressing shRNA to hMARCH8 (shRNA-M8): FW hM8sh1-5’- CCGGAAGACACTGGAGCAGAAATCACTCGAGTGATTTCTGCTCCAGTGTCTTTTTTTG-3’/RE hM8sh1-5’- AATTCAAAAAAAGACACTGGAGCAGAAATCACTCGAGTGATTTCTGCTCCAGTGTCTT-3’ for geneticin (G418) selection.

Lentiviruses were generated using shRNA-M2, shRNA-M8 or negative control vector containing scramble shRNA (shRNA-Ctrl; Addgene, 1864 and 13425) as per the manufacturer’s instruction and stored at −80°C for future experiments. Lentiviruses were used to transduce H9 and THP-1 cells at a MOI of 0.01. Cells were maintained in culture media supplemented with 5 μg/ml puromycin or 250 μg/ml G-418 (Research Products International) followed by limiting dilution. Before infection, PMA-differentiated lentivirus transduced THP-1 cells were treated with Vpx-containing SIV VLPs [33] in an 1:10 volume and maintained for 24 hours. Knockdown verification of MARCH2 and 8 in puromycin/G-418 selected cells was performed by RT-PCR analysis (see *HIV-1 infection*, *interferon treatment and human MARCH2 expression analysis* section) as well as by immunoblotting (see *Transfection and Immunoblotting* section). The following primers were used to detect *human MARCH3*: 5’-TGTGGCAGCCTAGTGAATGG-3’/5’-TTGTCCCCAAGGTCCCTGTA-3’ and *human MARCH8*: 5′-CTCTCGCACTTCTATCACGCCA-3′/5′-AAGTGGAGGCTTCCTGTGCAGT-3′ [9]. Forty-eight hours post transduction, primary CD4+ T cells (on day 5 of activation) were selected with puromycin (5 μg/ml). After 3 days of puromycin selection, viable primary CD4+ T cells were purified by density gradient centrifugation on Ficoll-Paque Plus (GE Healthcare) and used for infection experiments. Primary MDMs (on day 7 of differentiation) were transduced with lentiviruses and maintained for 48 h post transduction. Lentivirus transduced primary MDMs were selected with puromycin (3 μg/ml) or G-418 (450 μg/ml) for 48 h. Vpx containing SIV- VLPs [33] were added to lentivirus transduced primary MDMs cultures in a 1: 10 volume and maintained in complete RPMI supplemented with puromycin (3 μg/ml) or geneticin (450 μg/ml) for another 24 h. Finally, viable primary MDMs were used for infection experiments after knockdown verification by RT-PCR analysis (see *HIV-1 infection*, *interferon treatment and human MARCH2 expression analysis* section).

H9 cells and primary CD4+ T cells stably expressing shRNA-M2 or shRNA-Ctrl were infected with HIV-1^NL4-3^ (MOI of 0.01) and maintained at a density of 5 × 10^4^ cells/well in a 96-well plate. PMA-differentiated THP-1 and primary MDMs (both at 0.24 × 10^5^ cells/well) were seeded in a 24- well plate for 24 h. Cells were then infected with either HIV-1^AD8^ or HIV-1^JR-CSF^ (MOI of 0.5 for THP-1 cells and MOI of 0.1 for primary MDMs). All infections were performed by spinoculation (see *BlaM-Vpr fusion assay* section) followed by washing to remove viral inoculum. Cells were harvested at 1-, 3-, 5-, and 7-days post infection. DNA isolation and qPCR was performed as mentioned above (see *HIV-1 infection*, *interferon treatment and human MARCH2 expression analysis* section). The following primers were used for HIV-1^NL4-3^ *Env*, HIV-1^JR-CSF^ *Env* and HIV-1^AD8^ *Env* detection: NL4-3 *Env*: 5’-TAAAGTGCACTGATTTGAAGAATGAT-3’/ 5’-ATCTCTTATGCTTGTGCTGATATTG-3’; JR-CSF *Env*: 5’-TGATAGTAGGAGGCTTGATAGGT-3’/5’-GAGGAGGGTCTGAAACGATAAG-3’; NLAD8 *Env*: 5’-TGTATGCCCCTCCCATCAGA -3’/ 5’-TCCAGGTCTAAAGGTCTCGGT-3’. Primers used to detect HIV-1^AD8^ *Nef* were the same as mentioned above (see *HIV-1 infection*, *interferon treatment and human MARCH2 expression analysis* section).

### Assay for HIV-1 envelope and endogenous MARCH2 contents in H9 and THP-1 cells

H9 cells (1.4 × 10^6^) stably expressing shRNA-M2 or shRNA-Ctrl were infected with HIV-1^NL4-3^ (MOI of 0.01) and maintained in 25 ml of complete RPMI for 4 days. PMA- differentiated THP-1 cells (1.0 × 10^6^) stably expressing shRNA-M2, shRNA-M8 or shRNA-M2/M8 or shRNA-Ctrl were treated with Vpx-containing SIV VLPs for 24 hours as mentioned above. The following day, THP-1 cells were infected with HIV-1^AD8^ (MOI of 0.5) and maintained in 1.5 ml of complete RPMI for 2 days. Culture supernatants were harvested and processed as mentioned above (see *Virus preparation* section). For determination of HIV-1 gp120 and gp41 levels in the virions, HIV-1 p24 normalized culture supernatants were resolved on SDS-PAGE as described above. To probe for endogenous human MARCH2 incorporated into virions, culture supernatants were pelleted by ultracentrifugation as described above (see *OptiPrep gradient separation* section) and normalized for p24 followed by SDS-PAGE and immunoblotting. Band intensity was determined by densitometry using ImageJ software (NIH) (see *Transfection and Immunoblotting* section).

### Cell surface staining and FACS analyses

Cell surface expression levels of NL4-3 Env and NL4-3 TM^TR^ Env were evaluated as described previously with some modifications [27]. Briefly, 293T cells (2.5 × 10^5^ /well) were seeded in a 12-well plate. Next day, cells were co-transfected with plasmids for NL4-3.Luc.R-E- (1 μg) and either NL4-3 Env (3 μg), NL4-3 TM^TR^ Env (2 μg) or empty vector. Cells were harvested 48 h post transfection, washed, incubated with 20 μg/ml of monoclonal anti-HIV-1 gp120 antibody VRC01 (NIH HIV Reagent Program, NIAID, NIH, ARP-12033) for 1 h at 4°C. Cells were washed 3 times with fluorescence-activated cell sorting (FACS) buffer (1× PBS containing 2% FBS) followed by incubation with Alexa Flour 633 goat anti-human IgG (1: 2000 dilution, Invitrogen). Finally, cells were washed, fixed in 2% paraformaldehyde for 10 min at 4°C, and evaluated on BD FACSCelesta followed by data analysis using FlowJo version 10.8.0.

Cell surface expression levels of CD4, CXCR4 or CCR5 on different cell lines (H9 and THP1) and primary cells (CD4+ T cells and MDMs) stably expressing shRNA-M2, shRNA-M8, both shRNAs or shRNA-Ctrl (see *shRNA knockdown of human MARCH2 and MARCH8 in cell lines and primary cells followed by HIV-1 infection* section) were performed by cell surface staining followed by FACS. Cells were incubated with 1: 40 dilution of antibodies (PE mouse anti-human CD4 [RPA-T4], Invitrogen; APC mouse anti-human CD184/CXCR4 [12G5, Invitrogen; APC mouse anti-human CD195/CCR5 [NP-6G4, Invitrogen) or isotype controls (PE mouse IgG1 kappa [P3.6.2.8.1, Invitrogen]; APC mouse IgG2a kappa [eBM2a, Invitrogen]) for 30 minutes at 4°C, washed, fixed and acquired on BD LSRFortessa followed by data analysis using FlowJo version 10.8.0.

### HIV-1 cell-to-cell transfer assay

For experiments using transfected 293T as donor cells, we treated 12-well plate with 0.01% poly-L-lysine solution (Sigma-Aldrich) for 30 min at 37°C, dried and seeded 293T cells (0.25 × 10^6^/well). Next day, cells were co-transfected with HIV Gag-iGFP plasmid (1 μg), pcDNA3.1 hMARCH2 (1 μg) or empty vector. The following day, target H9 cells were pre-labelled with 1 μM of CellTrace Far Red Cell Proliferation Kit (Invitrogen) as per the manufacturer’s instruction. Culture media from transfected 293T cells were removed and replenished with 1 ml of RPMI media containing 1 × 10^6^ target cells. After 16 h of coculture, cells were harvested, washed, fixed in 2% paraformaldehyde for 10 min at 4°C, and acquired on BD FACSCelesta followed by data analysis using FlowJo version 10.8.0.

To examine the effect of endogenous human MARCH2 on HIV-1 cell-to-cell transfer, we used H9 cells stably expressing shRNA-M2 or shRNA-Ctrl (see *shRNA knockdown of human MARCH2 and MARCH8 in cell lines and primary cells followed by HIV-1 infection* section) as donors. shRNA-Ctrl or shRNA-M2 H9 donor cells (1 × 10^6^) were infected with 684.5 ng p24 equivalent of VSV-G pseudotyped HIV Gag-iGFP by spinoculation as mentioned above. Target TZM-bl cells were pre-labelled with 2 μM of CellTrace Far Red Cell Proliferation Kit and seeded in a 12-well plate (1 × 10^6^ cell/well). Next day, infected donor cells were washed twice in complete RPMI media and cocultured with TZM-bl cells for 24 h. Cocultured cells were processed as mentioned above.

### Immunofluorescence

Samples were processed for immunofluorescence as previously described^10^. AD293 cells (0.1 × 10^6^ cells/well) were seeded on poly-L-lysine-treated 12-mm coverslips (Carolina). Next day, cells were co-transfected with HIV Gag-iGFP plasmid (450 ng) and pcDNA3.1 hMARCH2 (50 ng). The following day, target H9 cells (1 × 10^6^) were pre-labelled with 3 μM of CellTracker Voilet-BMQC dye (Invitrogen) as per the manufacturer’s instruction and cocultured with transfected AD293 cells for 2 h at 37°C. Cocultured cells were washed, fixed with 4% paraformaldehyde and permeabilized with 0.3% Triton X-100 (Fischer Scientific) for 10 min at RT. Cells were then blocked with blocking buffer (1× PBS containing 4% bovine serum albumin [Research Products International] and 0.075% Tween 20 [Research Products International]) for 1 h at RT followed by incubation with rabbit anti-Myc (human MARCH2) (1: 250 dilution; Cell Signaling Technology) and mouse anti-ICAM-1 (1:200 dilution; MA5407, Invitrogen) in blocking buffer overnight at 4°C. Cells were then stained with Alexa Fluor 647 goat anti-Rabbit IgG (1: 1500 dilution; Invitrogen) and Alexa Fluor 568 goat anti-Mouse IgG (1: 3000 dilution; Invitrogen) in blocking buffer for 1 h at RT, washed 3 times in 1× PBS and mounted in antifade mounting media (0.25% 1, 4-Phenylenediamine and 90% Glycerol in 1× PBS). Images were acquired using 63×/1.4 oil immersion objective with a Leica TCS SP8 confocal microscope (Leica; Buffalo Grove, IL). Multiple fields with contacts between transfected AD293 and CellTracker Voilet BMQC- labelled H9 cells were selected from three independently performed experiments. For each field, a Z-series of images was acquired. Deconvolution and brightness adjustment were performed as previously described [71]. Line profile analyses were performed using Leica LAS X 3.7.4.23463 software. To examine co-localization of human MARCH2 with HIV-1 Gag, AD293 cells were co-transfected with HIV Gag-iGFP plasmid (450 ng) and pcDNA3.1 hMARCH2 (50 ng). Cells were processed as mentioned above using rabbit anti-Myc and Alexa Fluor 594 chicken anti-Rabbit IgG, (1: 2000 dilution; Invitrogen). Images were acquired using 63×/1.4 oil immersion objective with a Leica TCS SP8 confocal microscope (Leica; Buffalo Grove, IL) as mentioned above. Co-localization analyses were performed with a region of interest defined by Gag expression using the ImageJ (FIJI) Coloc2 plugin. To examine the localization of human MARCH2 WT and MARCH2 TM2^TR^ mutant when overexpressed in the absence of infection, AD293 cells were transfected with 50 ng of either pcDNA3.1 hMARCH2 WT or pcDNA3.1 MARCH2 TM2^TR^ and processed as mentioned above using rabbit anti-Myc and Alexa Fluor 594 chicken anti-Rabbit IgG (1: 2000 dilution; Invitrogen). Cells were stained with DAPI (Thermo Fischer Scientific, 1 μg/ml) for 10 minutes at RT, washed 3 times in 1× PBS and mounted in antifade mounting media. A Z-series of images were acquired using a 100×/1.46 Plan Apo oil immersion objective on a motorized Zeiss Axioimager M2 microscope equipped with an Orca ER charge-coupled-device (CCD) camera (Hamamatsu, Bridgewater, NJ), processed using Volocity (version 6.1, Acquisition Module (Improvision Inc., Lexington, MA)), and deconvolved by a constrained iterative algorithm using the Volocity Restoration Module.

### PCR analyses for MARCH2 isoforms

To detect isoforms of human MARCH2, PCR was performed using cDNAs from H9 and THP-1 cells and the following primers: hM2 isoform 1 (5’-CTCACAGAGTGGCTGAAGG-3’/ 5’-CGTCCAGAGGACATAGATGG-3’, hM2 isoform 2 (5’-CTCACAGAGGTCTCCTTCC-3’/5’- GTCTCCTCTGCCACCTTC-3’). Primers for GAPDH were also used as controls (see *HIV-1 infection*, *interferon treatment and human MARCH2 expression analysis* section).

### Statistical analyses

Statistical analyses were performed using GraphPad Prism software version 10.0. The statistical tests used to determine significance are described in the figure legends. A difference was considered to be significant for P values of ˂ 0.05.

## Supporting information

S1 Fig Confirmation of type I IFN response and HIV-1 infection in various cell lines. *ISG15* fold expression change relative to mock, normalized to GAPDH in **(A)** PMA-differentiated THP-1, H9, and 293T cells **(B)** primary CD4+ T cells and **(C)** MDMs from 3 donors treated with human IFN-β (500 units/ml) for 4 h, 8 h, 16 h, and 24 h. **(D)** HIV-1^JR-CSF^ and **(E)** HIV-1^NL4-3^ DNA levels relative to GAPDH in **(D)** PMA-differentiated THP-1 cells and **(E)** H9 cells at 4 h, 8 h, 16 h, and 24 h post infection (hpi). Mock indicates mock-treated (PBS). Graphs in **A**, **D** and **E** represent mean ± SEM from 3 independent experiments. Graphs in **B** and **C** represent mean ± SEM from 2 independent experiments.
(TIF)

S2 Fig Mouse MARCH2 C61G has no anti-HIV-1 function. **(A)** Schematic diagram showing MARCH2 (M2) chimeras (hMARCH2^N^-mMARCH2^C^ and mMARCH2^N^-hMARCH2^C^) generated by swapping the N- and C- terminal cytoplasmic tails between the human M2 (hM2) and mouse M2 (mM2). The positions of differing amino acid residues in human (orange) and mouse (blue) M2 are indicated. **(B)** NL4-3 Env pseudotyped luciferase reporter viruses were generated in the presence of wild type (WT) hM2, mM2, mM2 C61G mutant or empty vector (E.V.). Cells were infected and luciferase levels were measured 48 hpi followed by normalization to HIV-1 p24 levels of the input virus. The percentage (%) of relative infectivity was determined with respect to virus produced in the presence of E.V. Graphs represent means ± SD from 3 independent experiments. Statistical significance was determined by unpaired t-test (two-tailed). ns, non-significant; **, *P* ≤ 0.01. **(C)** MARCH2 G61C mutant retains its E3- ligase activity. Cell lysates from 293T cells co-transfected with plasmids expressing FLAG-tagged NEMO, HA-tagged Ubiquitin along with either M2 WT, M2 G61C mutant, or M2 W97A mutant were immunoprecipitated (IPed) with an anti-FLAG antibody followed by western blots probing with anti-Myc (M2), anti-HA (Ubiquitin), anti-FLAG (NEMO) and anti-β-Actin antibodies. Representative blot images from 3 independent experiments are shown. **(D)** AlphaFold predictions of hMARCH2 and hMARCH2-G61C visualized with iCn3D. The predicted structure of wild type M2 (left), and the structure of M2 G61C mutant (right). Both models represent the highest predicted confidence scores from AlphaFold and were visualized using the iCn3D web-based tool for structural analysis. (Transmembrane domain, TM; Extra cellular matrix, ECM; PDZ binding motif, PDZ motif; Ubiquitin, Ub).
(TIF)

S3 Fig Naturally occurring MARCH2 polymorphisms restrict HIV-1 envelope. NL4-3 Env pseudoviruses were produced in the presence of increasing concentrations of either wild type (WT) MARCH2 (M2), the MARCH2 polymorphic variants (M2 A54T, M2 R219P) or empty vector (E.V.). Subsequently, pseudoviruses were harvested and used for either **(A)** western blots were performed probing with anti-gp120, anti-gp41, anti-p24, anti-Myc (M2) and anti-β-Actin antibodies, or **(B)** for infectivity assays, during which luciferase levels were measured 48 hpi and normalized to HIV-1 p24 levels of the input virus. The percentage (%) of relative infectivity was determined with respect to virus produced in the presence of E.V. In **A**, representative gel images from 4 independent experiments are shown. In **B**, graphs represent mean ± SD from n = 4 independent experiments. Statistical significance was determined by unpaired t-test (two-tailed). ns, non-significant; *, *P* ≤ 0.05; **, *P* ≤ 0.01.
(TIF)

S4 Fig MARCH2 mutants localize to cellular membranes. Membrane fractionation of cells transfected with either empty vector (E.V.), wild type human MARCH2 (M2 WT) or various MARCH2 mutants: **(A)** PDZ binding motif (M2 PDZ^mut^) mutant, RING-CH domain (M2 RING-CH^mut^) mutant, N- terminal amino acid residues deletion (M2 Δ2–30; M2 Δ31–56) mutants followed by western blots. **(B)** western blots of cells transfected with either M2 WT, MARCH2 with either the N-terminal (TM1) or C-terminal (TM2) transmembrane domain swapped with those of human MARCH4 (M2 TM1^M4^, M2 TM2^M4^). **(C)** Membrane fractionation of cells transfected with either M2 WT, M2 TM1^M4^ or M2 in which the TM2 has been replaced with the TM domain of the human transferrin receptor (M2 TM2^TR^) followed by western blots. **(D)** M2 TM2^TR^ mutant and M2 WT have similar subcellular localization. AD293 cells transfected with M2 WT and M2 TM2^TR^ mutant expressing plasmids were subjected to immunostaining. Western blots in **A-C** were probed with anti-Myc (M2), anti-β-Actin and anti-GAPDH antibodies. Membrane fraction purity was verified by probing for GAPDH. In **A** and **C**, representative gel images from 2 independent experiments and in **B** representative images from 3 independent experiments are shown. In **D**, images were acquired from multiple fields from 3 independent experiments and representative deconvolved single Z-section images are shown. (membrane fraction, MF; cellular fraction, CF).
(TIF)

S5 Fig Isoforms of human MARCH2. Schematic diagram of human MARCH2 (M2) isoforms. The left panel represents the canonical (long) isoform of M2 (MARCH2-001) that contains two transmembrane domains (TM1 and TM2) and is 246 amino acids long. The right panel represents the non-canonical (short) isoform of M2 (MARCH2-002), which lacks both TM domains (Δ125–194 residues from canonical isoform) and consists of 176 amino acids. (Transmembrane domain, TM; Extra cellular matrix, ECM; PDZ binding motif, PDZ motif).
(TIF)

S6 Fig Replacing the transmembrane domain of NL4-3 envelope with that of the human Transferrin receptor (TR) does not affect its localization at the plasma membrane. Histogram of surface stain of 293T cells expressing either NL4-3 Env, NL4-3 TM^TR^ Env or empty vector (E.V.). Cells were harvested 48 h post transfection, washed, incubated with a monoclonal anti-HIV-1 gp120 antibody VRC01 and then subjected to FACS. Representative histogram from 3 independent experiments is shown.
(TIF)

S7 Fig Elucidating the effect of MARCH2 in released virions. **(A)** HIV-1 virion associated gp120 band intensities normalized to p24 band intensities relative to empty vector (E.V.) based on western blot images from Fig 7A. **(B)** HIV-1 virion associated gp120 or gp41 band intensities normalized to p24 band intensities relative to empty vector (E.V.) from western blot images of Fig 7C. **(C)** HIV-1 virion associated gp120 or gp41 band intensities normalized to p24 band intensities relative to empty vector (E.V.) from western blot images of Fig 7E. **(D)** Mouse MARCH2 (mM2) has no effect on HIV-1 gp120 and gp41 levels but is incorporated into virions. NL4-3 Env pseudotyped luciferase reporter viruses were produced in the presence of either wild type mM2, mM2 RING-CH^mut^ or E.V. Culture media and cell lysates were harvested and analyzed by western blots. **(E)** HIV-1 virion associated gp120 or gp41 band intensities normalized to p24 band intensities relative to empty vector (E.V.) conditions from western blot images from Fig 7H. In **A**, **B**, **C** and **E**, graphs represent mean ± SD from n = 3 independent experiments. In **D**, representative gel images from n = 3 independent experiments are shown. Statistical significance was determined using one-sample t-test (two-tailed) in **A**, **B** and **C**. In **E**, statistical significance was determined using one-sample t-test (two-tailed) when comparing with E.V. and unpaired t-test (two-tailed) between non E.V. conditions. ns, non-significant; **, *P* ≤ 0.01; ***, *P* ≤ 0.001.
(TIF)

S8 Fig Surface expression of CD4, CXCR4 and CCR5 receptors in MARCH2 knocked down cells and verification of MARCH2 knockdown in primary cells. **(A)** H9 cells stably expressing shCtrl or shM2 were stained with either PE-labelled anti-CD4, APC-labelled anti-CXCR4 or respective isotype controls and subjected to FACS. **(B)** Revisualization of the graph from Fig 8B with a focus on the earlier time points (24h and 48h) of H9 cells treated with either shCtrl or shM2 followed by HIV-1 infection. **(C)** THP-1 cells stably expressing shCtrl or shM2, shM8, both shM2 and shM8 were stained with either PE-labelled anti-CD4, APC-labelled anti-CCR5 or respective isotype controls and subjected to FACS. **(D)** PMA-differentiated THP-1 cells expressing shCtrl or shM2, shM8, both shM2 and shM8 were infected with HIV-1^JR-CSF^ and infected cells were harvested at the indicated time points. HIV-1 *nef* and *env* DNA levels were determined by RT-PCR and normalized to GAPDH. **(E)** *MARCH2 (M2)* fold expression change in shM2 treated cells relative to shCtrl expressing cells, normalized to GAPDH in primary human CD4+ T cells from 3 donors. **(F)** Primary human CD4+ T cells transduced with lentiviruses expressing shCtrl or shM2 were stained with either PE-labelled anti-CD4, APC-labelled anti-CXCR4 or respective isotype controls and subjected to FACS. **(G)** *M2* and *MARCH8* (M8) fold expression changes in shM2, shM8 or both shM2 and shM8 treated cells relative to shCtrl expressing cells, normalized to GAPDH in primary human MDMs from 2 donors. In **A** and **C**, representative histograms from two independent experiments are shown. In **F** and **H**, histograms from representative donor cell populations shown in Fig 8I and 8J are shown. Similar results were acquired from the other donors. Graphs in **B**, **D**, **E** and **G** represent mean ± SD (n = 3). Statistical significance was determined using unpaired t-test (two-tailed) in **B** and **D**, one-sample t-test (two-tailed) in **E**. In **G**, statistical significance when comparing with shCtrl were performed using one-sample t-test (two-tailed) and unpaired t-test (two-tailed) was used for comparisons among shM8 and shM2/M8. ns, non-significant; *, *P* ≤ 0.05; **, *P* ≤ 0.01; ***, *P* ≤ 0.001; ****, *P* ≤ 0.0001.
(TIF)

S9 Fig Confirmation for similar levels of HIV-1 expression in transfected or infected donor cells used in coculture experiments. **(A)** FACS plots of 293T cells co-transfected with plasmids for HIV Gag-iGFP along with either MARCH2 (M2) or empty vector (E.V.). **(B)** Western blots for MARCH2 (M2) levels and loading control β-Actin of cell lysates from **A**. **(C)** FACS plots of H9 cells stably expressing either shCtrl or shM2 and infected with VSV-G pseudotyped HIV Gag-iGFP virus. Shown are representative FACS plots or immunoblot images from 3 independent experiments. Mock (untransfected) 293T cells and uninfected shCtrl or shM2 expressing H9 cells were used for gating.
(TIF)

S1 Table The SNP frequencies of *MARCH2* at A54T (rs1133893) and R219P (rs34099346) obtained from 1000 Genomes and ranked by alternative allele frequencies of each population (release version: 20230706150541). (DOCX)

S2 Table Primers used for generating MARCH2 variants. (DOCX)

S1 Data Raw data used in this manuscript. (XLSX)
