# Supplementary figures and images for: MARCH2, a T cell specific factor that restricts HIV-1 infection
Source: PLoS Pathog. 2024 Jul 29;20(7):e1012330. doi: 10.1371/journal.ppat.1012330 (PMC11309421; doi:10.1371/journal.ppat.1012330)

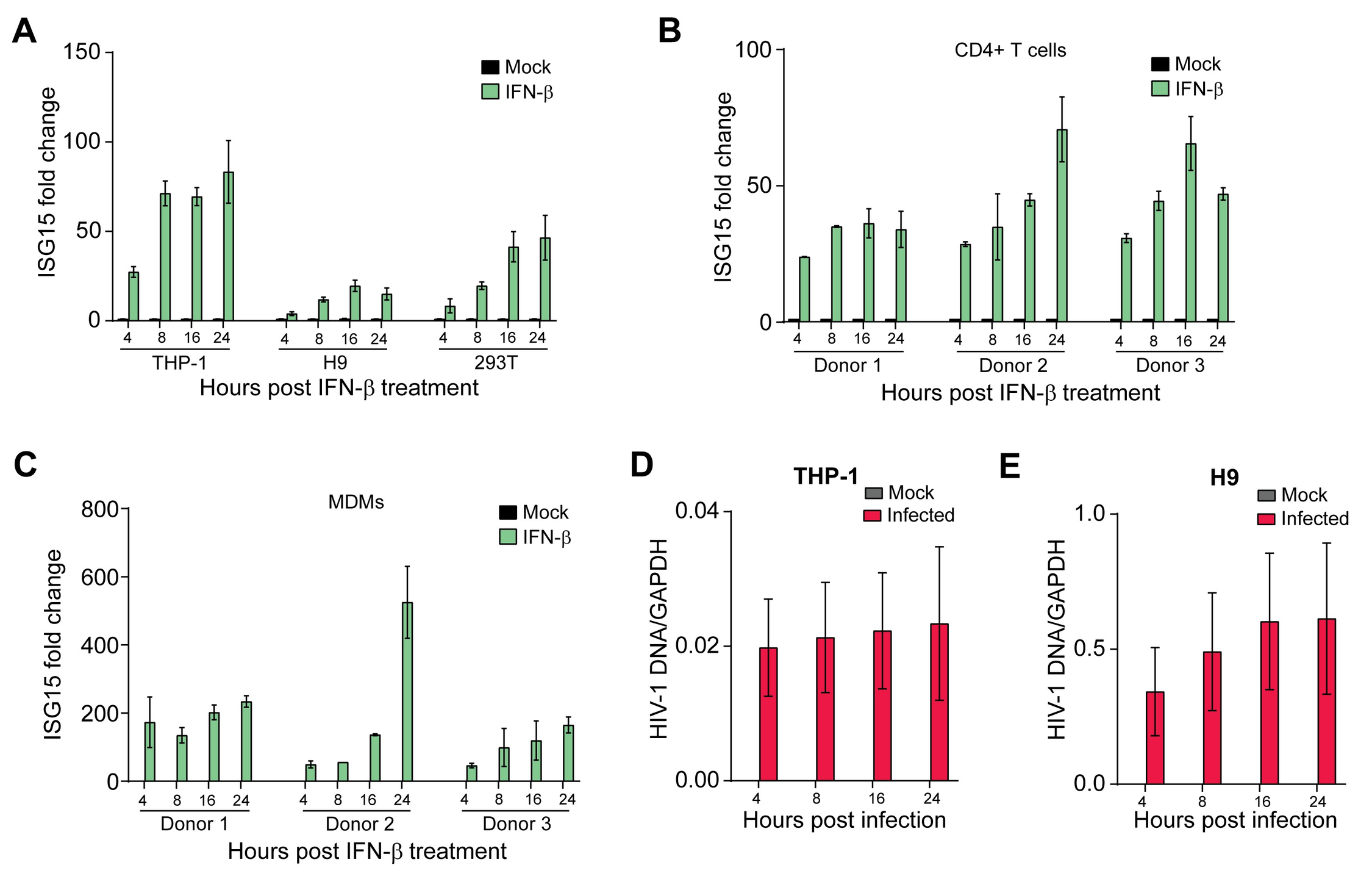

Supplement: S1 Fig — ISG15 fold expression change relative to mock, normalized to GAPDH in (A) PMA-differentiated THP-1, H9, and 293T cells (B) primary CD4+ T cells and (C) MDMs from 3 donors treated with human IFN-β (500 units/ml) for 4 h, 8 h, 16 h, and 24 h. (D) HIV-1JR-CSF and (E) HIV-1NL4-3 DNA levels relative to GAPDH in (D) PMA-differentiated THP-1 cells and (E) H9 cells at 4 h, 8 h, 16 h, and 24 h post infection (hpi). Mock indicates mock-treated (PBS). Graphs in A, D and E represent mean ± SEM from 3 independent experiments. Graphs in B and C represent mean ± SEM from 2 independent experiments. (TIF) [file ppat.1012330.s001.tif]

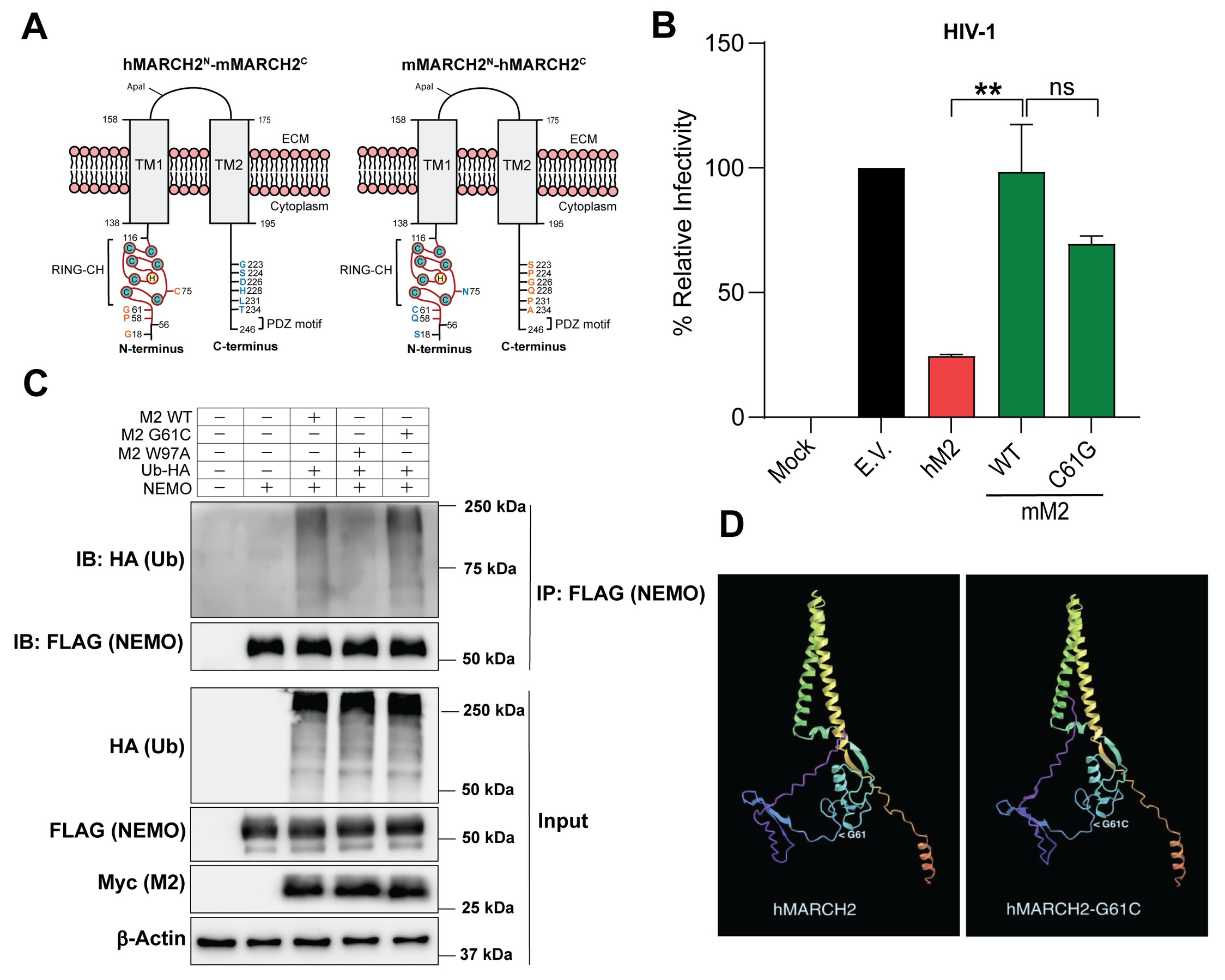

Supplement: S2 Fig — (A) Schematic diagram showing MARCH2 (M2) chimeras (hMARCH2N-mMARCH2C and mMARCH2N-hMARCH2C) generated by swapping the N- and C- terminal cytoplasmic tails between the human M2 (hM2) and mouse M2 (mM2). The positions of differing amino acid residues in human (orange) and mouse (blue) M2 are indicated. (B) NL4-3 Env pseudotyped luciferase reporter viruses were generated in the presence of wild type (WT) hM2, mM2, mM2 C61G mutant or empty vector (E.V.). Cells were infected and luciferase levels were measured 48 hpi followed by normalization to HIV-1 p24 levels of the input virus. The percentage (%) of relative infectivity was determined with respect to virus produced in the presence of E.V. Graphs represent means ± SD from 3 independent experiments. Statistical significance was determined by unpaired t-test (two-tailed). ns, non-significant; **, P ≤ 0.01. (C) MARCH2 G61C mutant retains its E3- ligase activity. Cell lysates from 293T cells co-transfected with plasmids expressing FLAG-tagged NEMO, HA-tagged Ubiquitin along with either M2 WT, M2 G61C mutant, or M2 W97A mutant were immunoprecipitated (IPed) with an anti-FLAG antibody followed by western blots probing with anti-Myc (M2), anti-HA (Ubiquitin), anti-FLAG (NEMO) and anti-β-Actin antibodies. Representative blot images from 3 independent experiments are shown. (D) AlphaFold predictions of hMARCH2 and hMARCH2-G61C visualized with iCn3D. The predicted structure of wild type M2 (left), and the structure of M2 G61C mutant (right). Both models represent the highest predicted confidence scores from AlphaFold and were visualized using the iCn3D web-based tool for structural analysis. (Transmembrane domain, TM; Extra cellular matrix, ECM; PDZ binding motif, PDZ motif; Ubiquitin, Ub). (TIF) [file ppat.1012330.s002.tif]

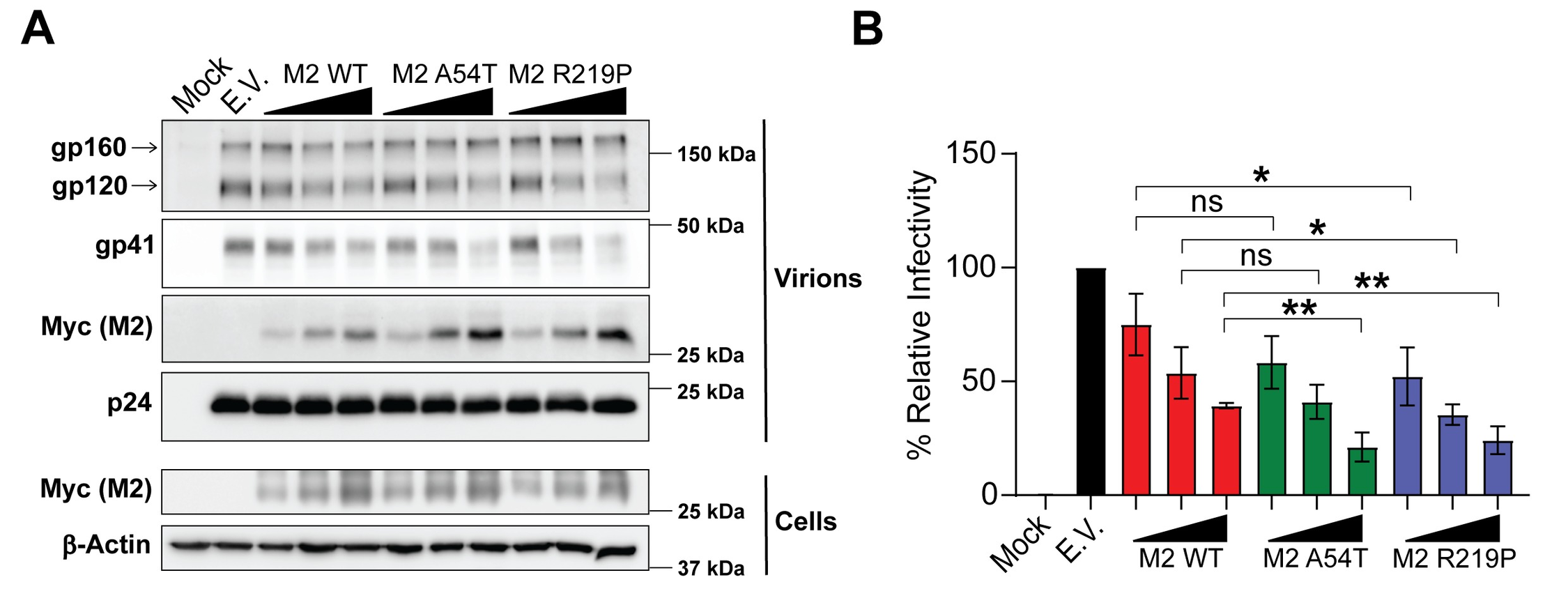

Supplement: S3 Fig — NL4-3 Env pseudoviruses were produced in the presence of increasing concentrations of either wild type (WT) MARCH2 (M2), the MARCH2 polymorphic variants (M2 A54T, M2 R219P) or empty vector (E.V.). Subsequently, pseudoviruses were harvested and used for either (A) western blots were performed probing with anti-gp120, anti-gp41, anti-p24, anti-Myc (M2) and anti-β-Actin antibodies, or (B) for infectivity assays, during which luciferase levels were measured 48 hpi and normalized to HIV-1 p24 levels of the input virus. The percentage (%) of relative infectivity was determined with respect to virus produced in the presence of E.V. In A, representative gel images from 4 independent experiments are shown. In B, graphs represent mean ± SD from n = 4 independent experiments. Statistical significance was determined by unpaired t-test (two-tailed). ns, non-significant; *, P ≤ 0.05; **, P ≤ 0.01. (TIF) [file ppat.1012330.s003.tif]

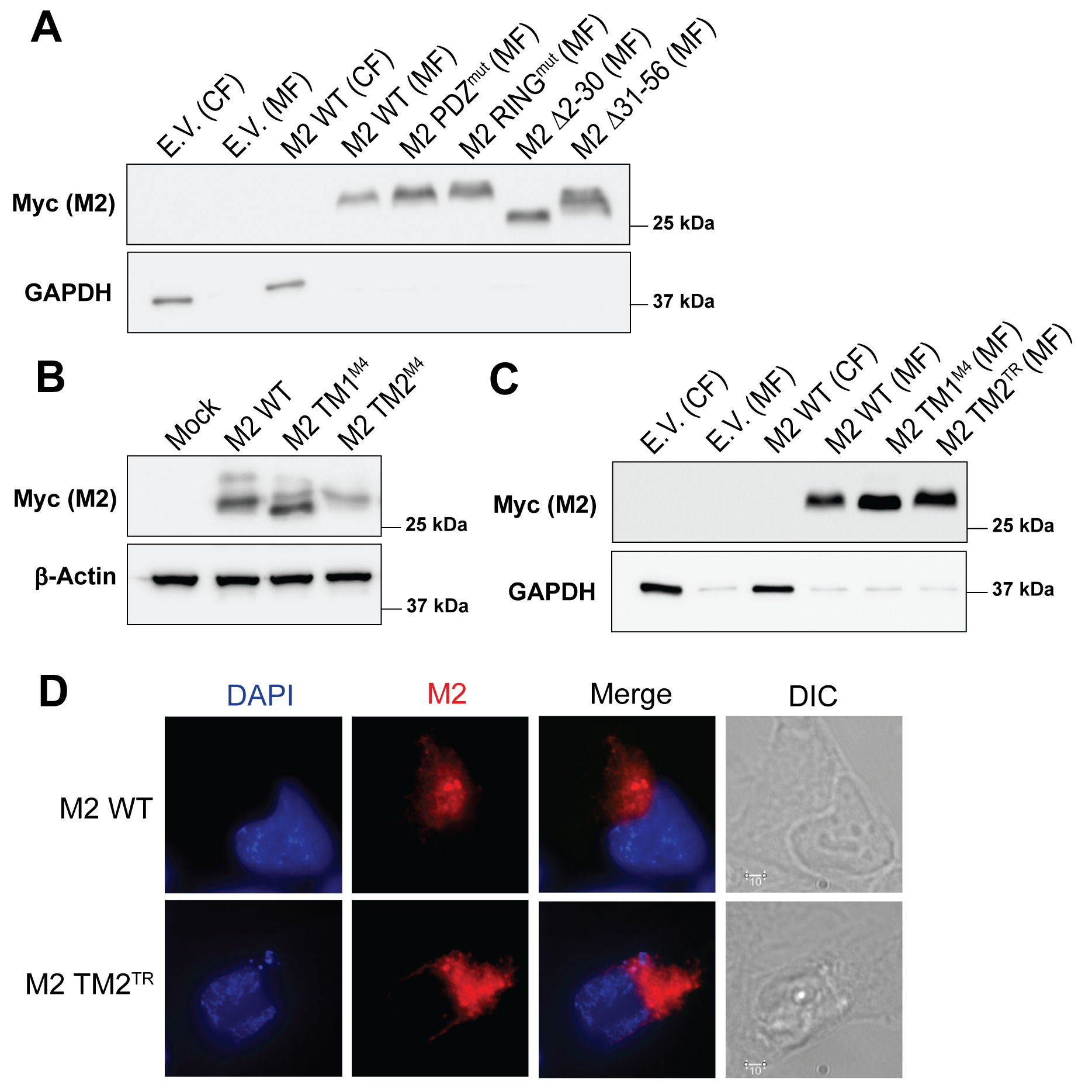

Supplement: S4 Fig — Membrane fractionation of cells transfected with either empty vector (E.V.), wild type human MARCH2 (M2 WT) or various MARCH2 mutants: (A) PDZ binding motif (M2 PDZmut) mutant, RING-CH domain (M2 RING-CHmut) mutant, N- terminal amino acid residues deletion (M2 Δ2–30; M2 Δ31–56) mutants followed by western blots. (B) western blots of cells transfected with either M2 WT, MARCH2 with either the N-terminal (TM1) or C-terminal (TM2) transmembrane domain swapped with those of human MARCH4 (M2 TM1M4, M2 TM2M4). (C) Membrane fractionation of cells transfected with either M2 WT, M2 TM1M4 or M2 in which the TM2 has been replaced with the TM domain of the human transferrin receptor (M2 TM2TR) followed by western blots. (D) M2 TM2TR mutant and M2 WT have similar subcellular localization. AD293 cells transfected with M2 WT and M2 TM2TR mutant expressing plasmids were subjected to immunostaining. Western blots in A-C were probed with anti-Myc (M2), anti-β-Actin and anti-GAPDH antibodies. Membrane fraction purity was verified by probing for GAPDH. In A and C, representative gel images from 2 independent experiments and in B representative images from 3 independent experiments are shown. In D, images were acquired from multiple fields from 3 independent experiments and representative deconvolved single Z-section images are shown. (membrane fraction, MF; cellular fraction, CF). (TIF) [file ppat.1012330.s004.tif]

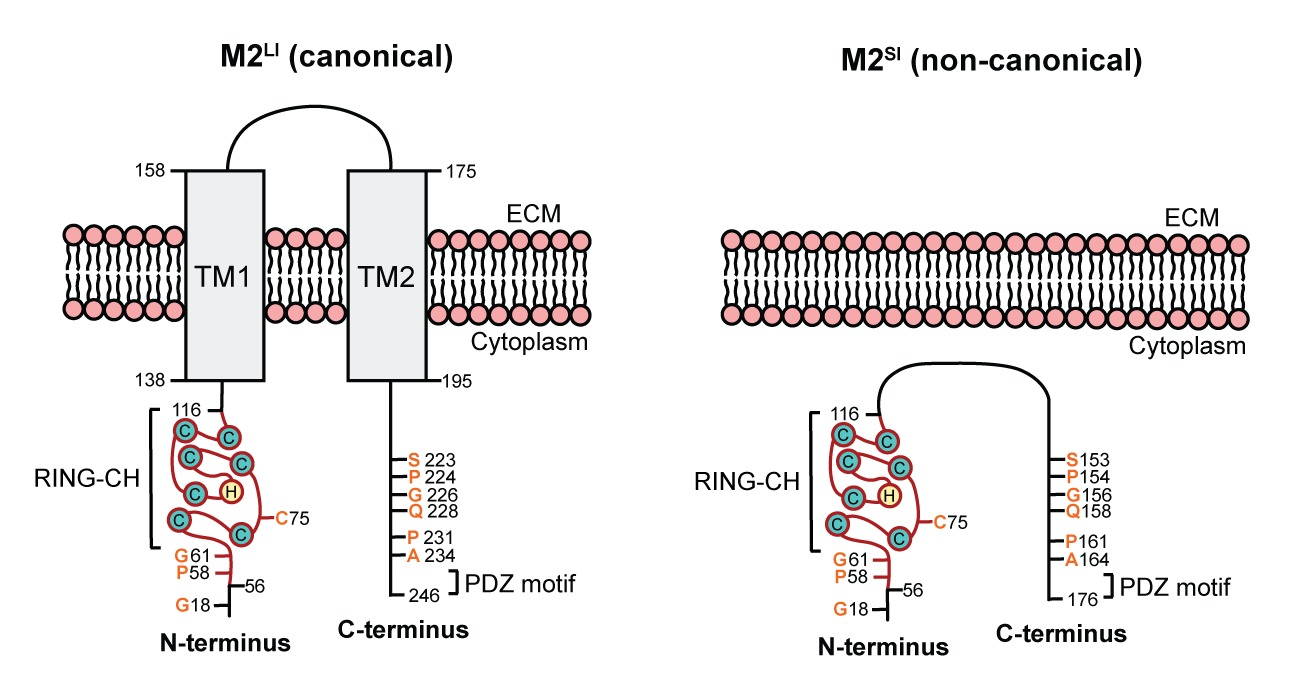

Supplement: S5 Fig — Schematic diagram of human MARCH2 (M2) isoforms. The left panel represents the canonical (long) isoform of M2 (MARCH2-001) that contains two transmembrane domains (TM1 and TM2) and is 246 amino acids long. The right panel represents the non-canonical (short) isoform of M2 (MARCH2-002), which lacks both TM domains (Δ125–194 residues from canonical isoform) and consists of 176 amino acids. (Transmembrane domain, TM; Extra cellular matrix, ECM; PDZ binding motif, PDZ motif). (TIF) [file ppat.1012330.s005.tif]

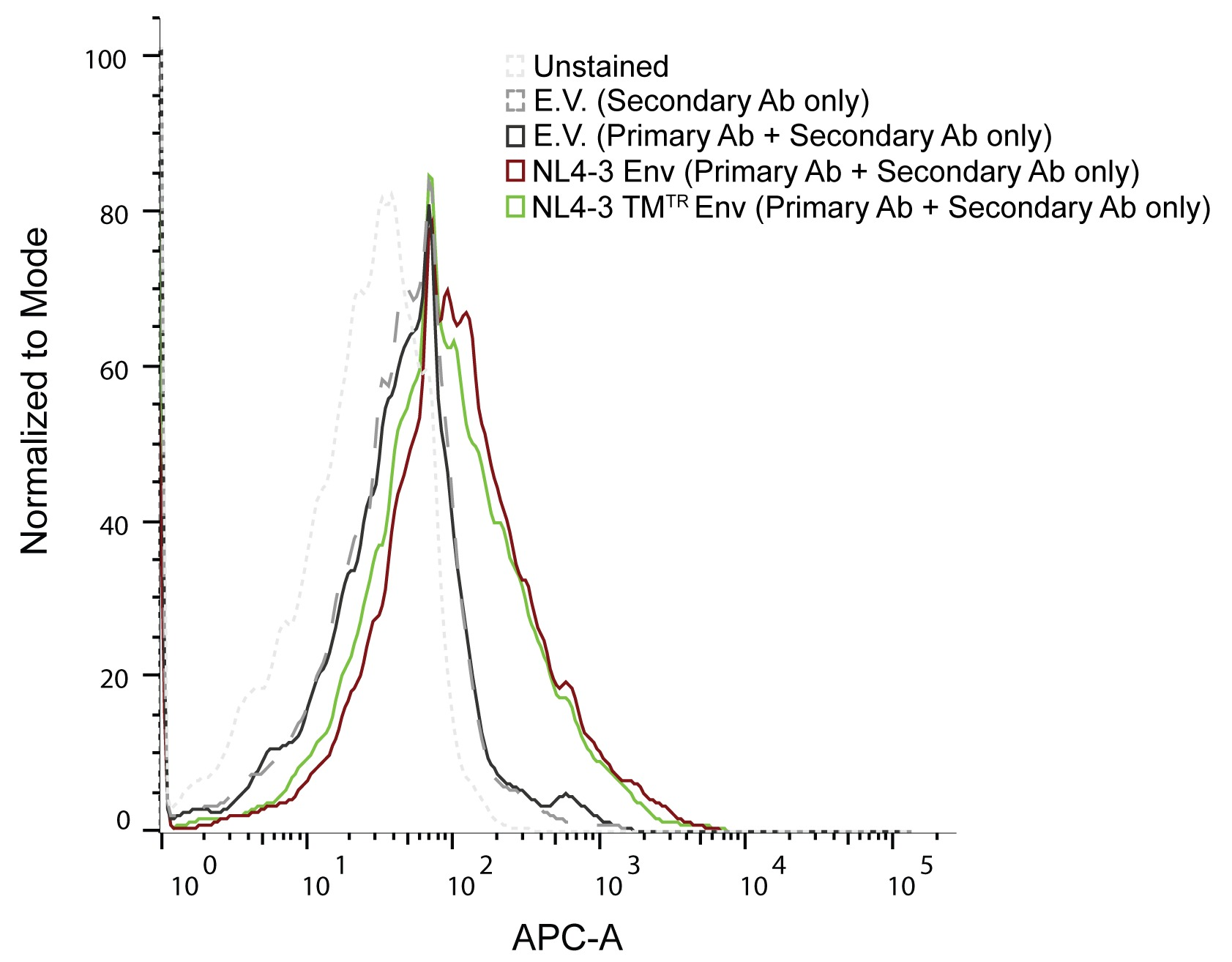

Supplement: S6 Fig — Histogram of surface stain of 293T cells expressing either NL4-3 Env, NL4-3 TMTR Env or empty vector (E.V.). Cells were harvested 48 h post transfection, washed, incubated with a monoclonal anti-HIV-1 gp120 antibody VRC01 and then subjected to FACS. Representative histogram from 3 independent experiments is shown. (TIF) [file ppat.1012330.s006.tif]

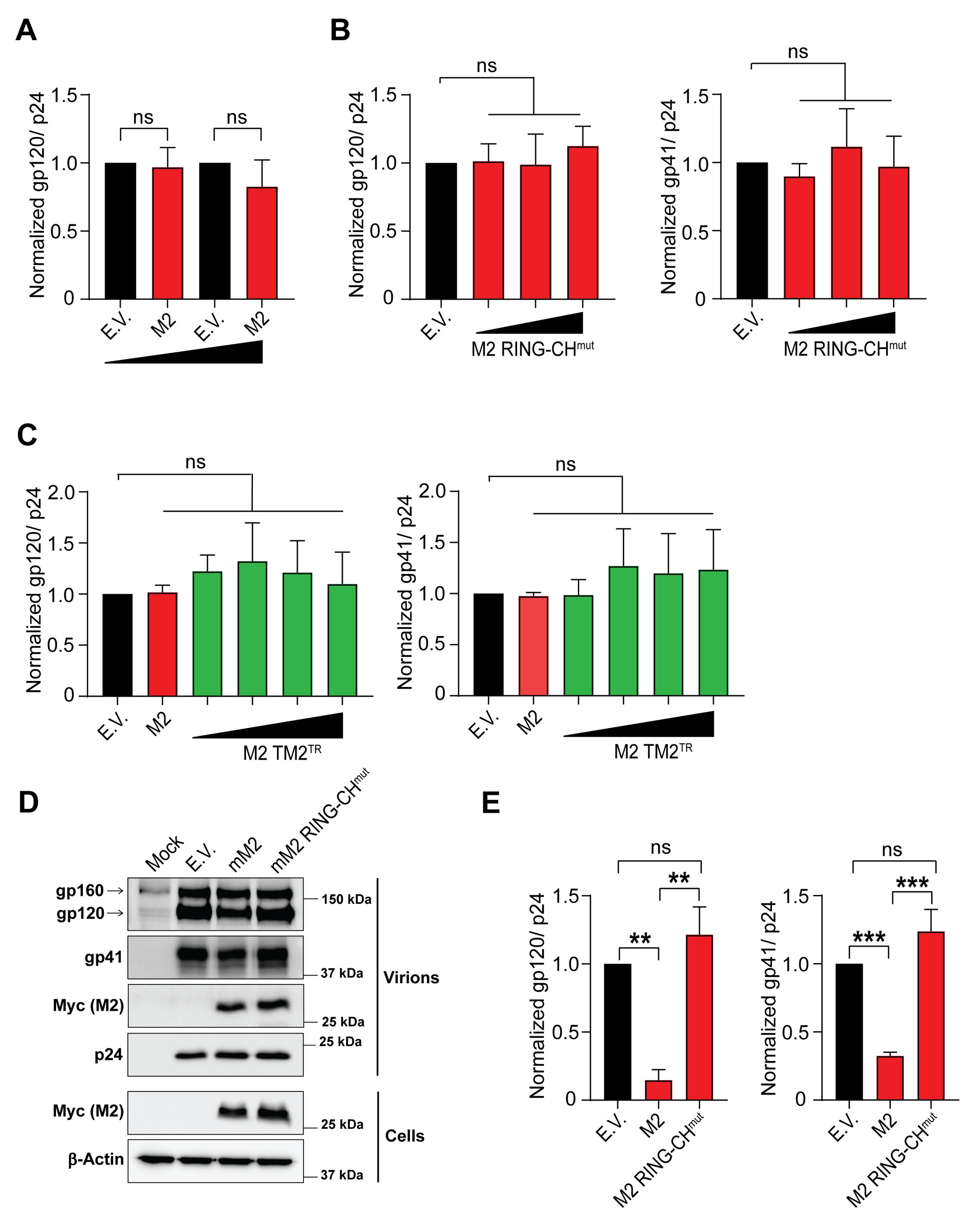

Supplement: S7 Fig — (A) HIV-1 virion associated gp120 band intensities normalized to p24 band intensities relative to empty vector (E.V.) based on western blot images from Fig 7A. (B) HIV-1 virion associated gp120 or gp41 band intensities normalized to p24 band intensities relative to empty vector (E.V.) from western blot images of Fig 7C. (C) HIV-1 virion associated gp120 or gp41 band intensities normalized to p24 band intensities relative to empty vector (E.V.) from western blot images of Fig 7E. (D) Mouse MARCH2 (mM2) has no effect on HIV-1 gp120 and gp41 levels but is incorporated into virions. NL4-3 Env pseudotyped luciferase reporter viruses were produced in the presence of either wild type mM2, mM2 RING-CHmut or E.V. Culture media and cell lysates were harvested and analyzed by western blots. (E) HIV-1 virion associated gp120 or gp41 band intensities normalized to p24 band intensities relative to empty vector (E.V.) conditions from western blot images from Fig 7H. In A, B, C and E, graphs represent mean ± SD from n = 3 independent experiments. In D, representative gel images from n = 3 independent experiments are shown. Statistical significance was determined using one-sample t-test (two-tailed) in A, B and C. In E, statistical significance was determined using one-sample t-test (two-tailed) when comparing with E.V. and unpaired t-test (two-tailed) between non E.V. conditions. ns, non-significant; **, P ≤ 0.01; ***, P ≤ 0.001. (TIF) [file ppat.1012330.s007.tif]

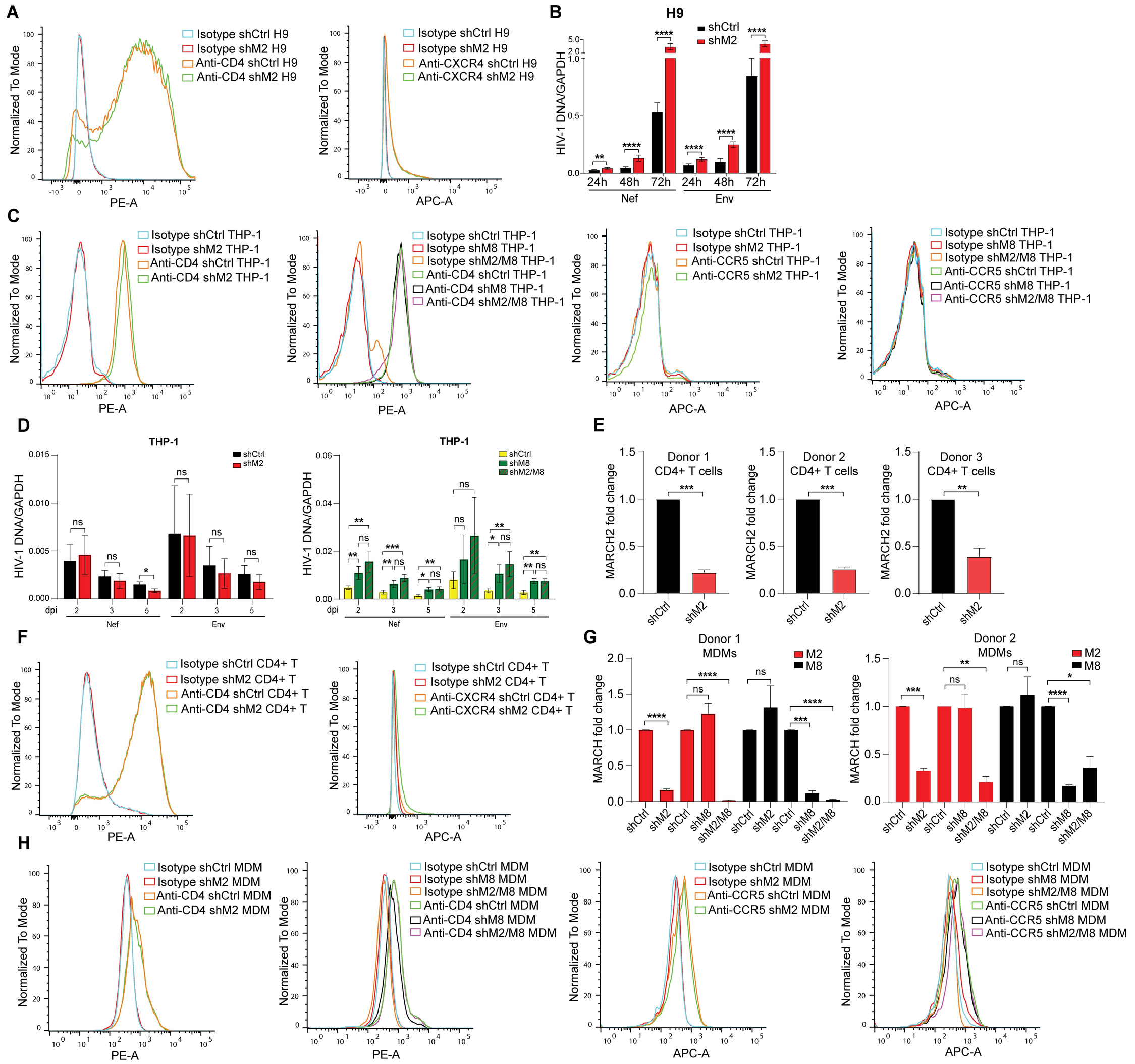

Supplement: S8 Fig — (A) H9 cells stably expressing shCtrl or shM2 were stained with either PE-labelled anti-CD4, APC-labelled anti-CXCR4 or respective isotype controls and subjected to FACS. (B) Revisualization of the graph from Fig 8B with a focus on the earlier time points (24h and 48h) of H9 cells treated with either shCtrl or shM2 followed by HIV-1 infection. (C) THP-1 cells stably expressing shCtrl or shM2, shM8, both shM2 and shM8 were stained with either PE-labelled anti-CD4, APC-labelled anti-CCR5 or respective isotype controls and subjected to FACS. (D) PMA-differentiated THP-1 cells expressing shCtrl or shM2, shM8, both shM2 and shM8 were infected with HIV-1JR-CSF and infected cells were harvested at the indicated time points. HIV-1 nef and env DNA levels were determined by RT-PCR and normalized to GAPDH. (E) MARCH2 (M2) fold expression change in shM2 treated cells relative to shCtrl expressing cells, normalized to GAPDH in primary human CD4+ T cells from 3 donors. (F) Primary human CD4+ T cells transduced with lentiviruses expressing shCtrl or shM2 were stained with either PE-labelled anti-CD4, APC-labelled anti-CXCR4 or respective isotype controls and subjected to FACS. (G) M2 and MARCH8 (M8) fold expression changes in shM2, shM8 or both shM2 and shM8 treated cells relative to shCtrl expressing cells, normalized to GAPDH in primary human MDMs from 2 donors. In A and C, representative histograms from two independent experiments are shown. In F and H, histograms from representative donor cell populations shown in Fig 8I and 8J are shown. Similar results were acquired from the other donors. Graphs in B, D, E and G represent mean ± SD (n = 3). Statistical significance was determined using unpaired t-test (two-tailed) in B and D, one-sample t-test (two-tailed) in E. In G, statistical significance when comparing with shCtrl were performed using one-sample t-test (two-tailed) and unpaired t-test (two-tailed) was used for comparisons among shM8 and shM2/M8. ns, non-significant; *, [file ppat.1012330.s008.tif]

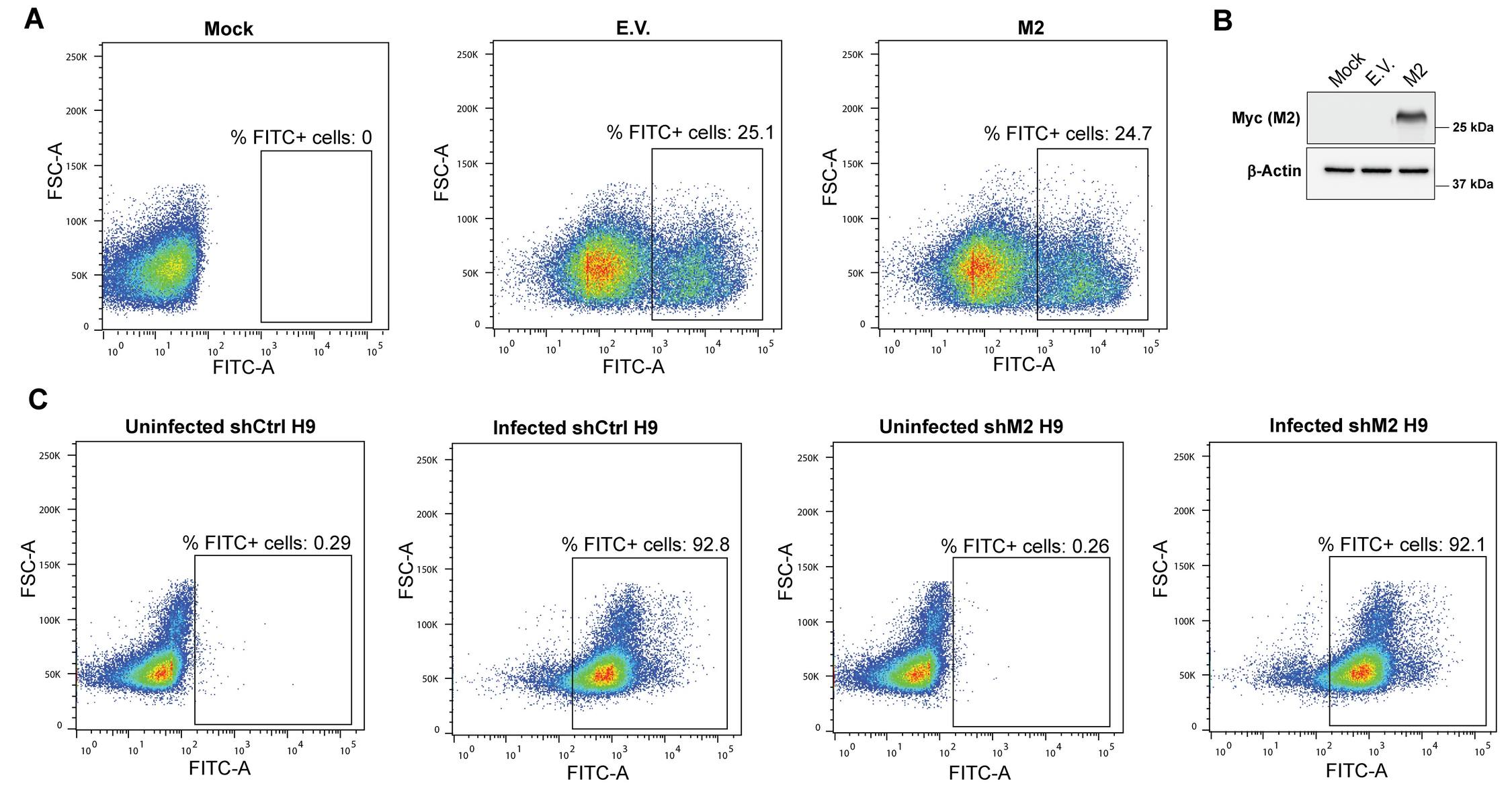

Supplement: S9 Fig — (A) FACS plots of 293T cells co-transfected with plasmids for HIV Gag-iGFP along with either MARCH2 (M2) or empty vector (E.V.). (B) Western blots for MARCH2 (M2) levels and loading control β-Actin of cell lysates from A. (C) FACS plots of H9 cells stably expressing either shCtrl or shM2 and infected with VSV-G pseudotyped HIV Gag-iGFP virus. Shown are representative FACS plots or immunoblot images from 3 independent experiments. Mock (untransfected) 293T cells and uninfected shCtrl or shM2 expressing H9 cells were used for gating. (TIF) [file ppat.1012330.s009.tif]
