# Supplementary material for: MARCH2, a T cell specific factor that restricts HIV-1 infection
Source: PLoS Pathog. 2024 Jul 29;20(7):e1012330. doi: 10.1371/journal.ppat.1012330 (PMC11309421; doi:10.1371/journal.ppat.1012330)
Supplement: S1 Table — (DOCX) [file ppat.1012330.s010.docx]

**S1 Table:** The SNP frequencies of *MARCH2* at A54T (rs1133893) and R219P (rs34099346) obtained from 1000 Genomes and ranked by alternative allele frequencies of each population (release version: 20230706150541)

| **SNVs** | **Population** | **Frequencies** | | **Sample Size** |
| --- | --- | --- | --- | --- |
|  |  | **Ref Allele** | **Alt Allele** |  |
| NP_001356706.1:p.Ala54Thr (rs1133893) | Global | G=0.8011 | A=0.1989 | 5008 |
|  | **European** | **G=0.7078** | **A=0.2922** | **1006** |
|  | **American** | **G=0.728** | **A=0.272** | **694** |
|  | East Asian | G=0.7619 | A=0.2381 | 1008 |
|  | South Asian | G=0.768 | A=0.232 | 978 |
|  | Africa | G=0.9652 | A=0.0348 | 1322 |
| NP_001356706.1:p.Ag219Pro (rs34099346) | Global | G=0.9065 | C=0.0935 | 5008 |
|  | **South Asian** | **G=0.826** | **C=0.174** | **978** |
|  | American | G=0.833 | C=0.167 | 694 |
|  | European | G=0.8330 | C=0.167 | 1006 |
|  | East Asian | G=0.9980 | C=0.0020 | 1008 |
|  | Africa | G=0.9909 | C=0.0091 | 1322 |
