# Supplementary material for: MARCH2, a T cell specific factor that restricts HIV-1 infection
Source: PLoS Pathog. 2024 Jul 29;20(7):e1012330. doi: 10.1371/journal.ppat.1012330 (PMC11309421; doi:10.1371/journal.ppat.1012330)
Supplement: S2 Table — (DOCX) [file ppat.1012330.s011.docx]

**S2 Table: Primers used for generating MARCH2 variants**

| **Constructs** | **Primers used** |
| --- | --- |
| pBJ5 mMARCH2 S18G | 5’-AGCCCTGCCTTTTCCAAGGTTGTAGAG-3’ and 5’-GCCCGAACAGTCACATAGGGAGCCAGG-3’ |
| pBJ5 mMARCH2 Q58P | 5’-AGTGACTGTCCCTTCTGCCGA-3’ and 5’- CGGTGAATCCAAAGCCCGGAT-3’ |
| pBJ5 mMARCH2 C61G | 5’-CCCTTCTGCCGAATCTGCC-3’ and 5’- ACCGTCACTCTGTGAATCCAA-3’ |
| pBJ5 mMARCH2 N75C | 5’-GCACTCCCCATTTGCTCC-3’ and 5’-TTGCTGTCCCCATGTGGC-3’ |
| pBJ5 hMARCH2 G18S | 5’-TCCAGCAGCCCTGCCTTC-3’ and 5’-GCAGTCACACAGGGAGCC-3’ |
| pBJ5 hMARCH2 P58Q | 5’-GGACACACAGAGTGATGGTCC-3’and 5’-AAGGCACGGATGACGGTG-3’ |
| pBJ5 hMARCH2 G61C | 5’-TGTCCTTTCTGCCGGATCTG-3’ and 5’-ATCACTCGGTGTGTCCAAGGC-3’ |
| pBJ5 hMARCH2 C75N | 5’-GGGGAGAACTTGCTGTCCC-3’and 5’-GTTCGCTCCCTCATGGCAG-3’ |
| pBJ5 hMARCH2 A54T | 5’-ACCTTGGACACACCGAGTGATG-3’ and 5’-ACGGATGACGGTGGAGAG-3’ |
| pBJ5 hMARCH2 R219P | 5’-AAGATCCCGGAGGCGGACA-3’ and 5’-CAGGCGAACTTTCTGGTTGG-3’ |
| pcDNA3.1 hMARCH2 W97A (RING-CH mutant) | 5’-GCTGTCTGGAGAAGGCGCTTTCCTCATC-3’ and 5’-TCTTATGCACGGCACCCAG-3’ |
| pcDNA3.1 hMARCH2 C64/67S | 5’-AGCCATGAGGGAGCGAACG-3'  5'-GATCCGGCTGAAAGGACCATCAC-3' |
| pcDNA3.1 hMARCH2 Δ2-30 | 5’-CATGGTACCAAGCTTAACTAG-3’ and 5’-GCCCGTAGCCTCCACGAC-3’ |
| pcDNA3.1 hMARCH2 Δ31-56 | 5’-CTCGGACCGCCCCAGTATGTG-3’ and 5’-ACACCGAGTGATGGTCCT-3’ |
| pcDNA3.1 hMARCH2 PDZ mutant | 5’-GCAGCAGCGGCCGCTCGAGTCTAG-3’ and 5’-CGCCGCCTCTGCCACCTTCTTCAG-3’ |
| pcDNA3.1 hMARCH2 TM1^M4^ | **hM4-TM1**: 5’-CGGACGGAGAAGCGGACAGTTGCAGCCGCCATCCTGGGCT-3’  and 5’-GGCCCCGCGCAGGCATGACCAGATGAGCCAAGAAATACTGG-3’  **hMARCH2 ΔTM1**: 5’-CTTGGCTCATCTGGTCATGCCTGCGCGGGGCCCAGGACC-3’  and 5’- GATGGCGGCTGCAACTGTCCGCTTCTCCGTCCGCGG-3’ |
| pcDNA3.1 hMARCH2 TM2^M4^ | **hM4-TM2**: 5’-CGGCTCCACAGCCAGCTCTTCCAGATCTGCTACGGGA-3’ and 5’- GCGGAAGGAGACCAGGATGATGAGACCTATGCACACC-3’  **hMARCH2 ΔTM2**: 5’- CATAGGTCTCATCATCCTGGTCTCCTTCCGCTACCAC-3’ and 5’-GCAGATCTGGAAGAGCTGGCTGTGGAGCCGGAGGTG-3’ |
| pcDNA3.1 hMARCH2 TM2^TR^ | **TFR-TM**: 5’-CACAGCCAGCTGGAGTATGGGACTATTGCTGTG-3’ and 5’-GTGGTAGCGGAAGGAATAGCCCAAGTAGCCAATC-3’  **hMARCH2 ΔTM2**: 5’-GGCTACTTGGGCTATTCCTTCCGCTACCACTGC-3’ and 5’- AGCAATAGTCCCATACTCCAGCTGGCTGTGGAG-3’ |
| pcDNA3.1 NL4-3 TM^TR^ Env | **NL4-3 ΔTM**: 5’-GGCTACTTGGGCTATAATAGAGTTAGGCAGGGA-3’ and 5’-AGCAATAGTCCCATATAATTTTATATACCACAGCCAA-3’  **TFRC TM**: 5’-TGGTATATAAAATTATATGGGACTATTGCTGTG-3’ and 5’-CCTGCCTAACTCTATTATAGCCCAAGTAGCCAATC-3’ |
| hMARCH2ApaIuncut | 5’-CCCGAGGGTCCCCAGCATTCTC-3’ and 5’-GCTGTCCGCCTCCCGGATCTTCAGG-3’ |
| pcDNA3.1 mMARCH2 W97A (RING-CH mutant) | 5’-GCTGCCTGGAGAAAGCGCTGTCTTCCTCC-3’  5’-TCTTGTGCACAGCTCCCAG-3’ |
